# Integrated Single‐Nucleus Multi‐Omics Atlases Reveal Lineage Plasticity and Regulatory Networks of Luminal Epithelial Cells During Mammary Gland Lactation and Involution

**DOI:** 10.1002/advs.76753

**Published:** 2026-07-27

**Authors:** Xiaoru Yan, Xiaoyu Mi, Guanghui Tan, Xinmei Li, Zhenliang Zhu, Zhenyu Wei, Huimei Fan, Yamei Wu, Tao Shi, Lingzhao Fang, Yuanpeng Gao, Yu Wang, Jun Liu

**Affiliations:** ^1^ College of Veterinary Medicine Shaanxi Centre of Stem Cells Engineering & Technology Northwest A&F University Yangling Shaanxi China; ^2^ College of Veterinary Medicine Key Laboratory of Animal Biotechnology of the Ministry of Agriculture Northwest Agriculture & Forestry University Yangling Shaanxi China; ^3^ Key Laboratory of Animal Genetics Breeding and Reproduction of Shaanxi Province College of Animal Science and Technology Northwest A&F University Yangling Shaanxi China; ^4^ State Key Laboratory of Molecular Biology Center For Excellence in Molecular Cell Science Shanghai Institute of Biochemistry and Cell Biology Chinese Academy of Sciences Shanghai China; ^5^ Center for Quantitative Genetics and Genomics Aarhus University Aarhus Denmark

**Keywords:** chromatin accessibility, luminal progenitors, mammary gland, milk production traits, snRNA & snATAC, spatial transcriptomics

## Abstract

Dynamic changes in mammary cells are essential for sustaining lactation and maintaining epithelial homeostasis. However, the phenotypic transition process of mammary cells during lactation remains unclear. Here, single‐nucleus RNA sequencing (snRNA‐seq) of 64 199 cells and single‐nucleus chromatin accessibility sequencing (snATAC‐seq) of 78 984 cells were generated from the goat mammary gland of dry and lactation stages. A total of 18 cell types were annotated, and spatial transcriptomic analysis confirmed the localization of lactation‐related cell types within the mammary tissue. Enrichment analysis of SNP within cell type‐specific chromatin accessibility regions revealed strong associations between mammary epithelial cells (MECs) with milk production traits. To further explore the MECs functional diversification during lactation and their differences from the dry stage, four differentiation trajectories from luminal progenitor to luminal mature cells were reconstructed. Lineage‐specific gene regulatory networks (GRNs) were constructed by integrating snRNA‐seq and snATAC‐seq data, and stage‐specific signals were identified through cell‐cell communications. Finally, to explore the evolutionary conservation and divergence of MECs, cross‐species comparative analyses were conducted and revealed MEC differential evolutionary rates, conserved milk‐producing subtypes, and lineage‐specific populations driving species‐specific differences in milk composition. Overall, these findings uncover the coordinated transcriptional and chromatin dynamics that drive mammary epithelial differentiation and functional maintenance during lactation.

## Graphical abstract

This study integrates single‐cell multi‐omics, spatial transcriptomics, and cross‐species comparative analyses to systematically characterize the cellular composition and differentiation trajectories of goat mammary epithelial cells, along with the gene regulatory networks and intercellular communication mechanisms governing these trajectories, thereby revealing their associations with milk production traits and the evolutionary features underlying species‐specific milk composition.

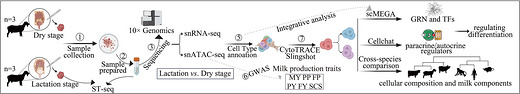

## 1 Introduction

The mammary gland is a complex exocrine organ that plays a critical role in providing essential nutrition, immune protection and developmental signals to offspring through lactation [1]. It undergoes cyclic remodeling in response to physiological changes during pregnancy, lactation and involution [2]. Mammary tissue consists of multiple specialized cell types, including estrogen receptor (ER)^−^ milk‐secreting cells (Lactocytes) responsible for milk synthesis and secretion; luminal hormone‐sensing (LHS) cells that rapidly respond to hormonal signal; basal‐myoepithelial (B‐Myo) cells that facilitate milk ejection and clearance, as well as diverse immune cells (e.g., macrophages) and stromal components (e.g., endothelial cells) that contribute to maintenance of structural integrity and immune homeostasis in the mammary microenvironment [3, 4, 5]. The dynamic balance between Lactocytes and LHS cells, which underlies the luminal epithelial cells, and the differentiation trajectories of these epithelial cells are closely associated with the regulation of lactation function [6]. Single‐cell/nucleus RNA sequencing (sc/snRNA‐seq) data have delineated the cellular hierarchy and lineage transitions in mammary glands of humans, mice and pigs across different lactation stages, revealing that Lactocytes and LHS cells are two distinct cell lineages derived from luminal progenitors [7, 8, 9, 10, 11, 12, 13, 14]. Mammary cell research has uncovered progenitor cells that not only facilitate mammary gland development and lactation but also include progenitors capable of generating diverse structural components of the gland [15]. The cellular diversity of the mammary gland and the high plasticity of mammary progenitor cells support the dynamic balance of cellular composition during the lactation cycle [16].

During the lactation initiation and cessation, mammary cells rapidly change identity alongside chromatin remodeling and cellular reorganization. This is particularly evident in livestock such as dairy goats, where after weaning, the mammary gland undergoes involution accompanied by extensive tissue remodeling to restore its baseline architecture and prepare for the next lactation cycle. Nonetheless, the cellular dynamics and regulatory mechanisms governing these processes remain largely unclear. Recent lineage tracing, ultrastructural and transcriptomic studies have revealed aspects of cellular lineages, organelle dynamics, molecular architecture, and functional regulation in ruminant mammary glands [17, 18, 19, 20]. However, compared with those in humans and model organisms, single‐cell studies on the mammary gland in ruminants (such as goats) remain limited, and systematic cross‐species and cross‐stage comparisons are still lacking [21]. The integrative analysis of single‐nucleus chromatin accessibility sequencing (snATAC‐seq) and sc/snRNA‐seq allows for unbiased clustering of cells across differentiation states based on shared transcriptomic and epigenomic signatures, enabling the construction of gene regulatory networks (GRNs) that regulate MECs differentiation [22]. Thus, leveraging snRNA‐seq and snATAC‐seq to characterize mammary cell dynamics across lactation is essential for elucidating the molecular programs driving mammary gland remodeling. Moreover, genome‐wide association studies (GWAS) on lactation traits have identified hundreds of genetic variants associated with milk yield (MY), protein percentage (PP), fat percentage (FP), protein yield (PY), fat yield (FY), and somatic cell score (SCS) [23, 24, 25]. However, the vast majority of these variants reside in non‐coding regions. Integrating GWAS results with single‐cell omics data enables to prioritize potential causal variants, genes, and cell types for these complex milk production traits, contributing to their genetic improvement program in goats [26].

Here, we performed snRNA‐seq and snATAC‐seq on mammary glands from six dairy goats, three at the dry stage and three at the lactation stage, to characterize cellular and regulatory changes during lactation. A total of 18 distinct cell types were identified, including seven epithelial subtypes, notably luminal adaptive secretory precursor 4 (LASP4) and three molecularly distinct Lactocyte populations. Spatial transcriptomics of lactating mammary tissue confirmed the organized architecture and heterogeneity of MECs, while snATAC‐seq revealed cell type‐specific chromatin accessibility landscapes. Integration of snATAC‐seq from the lactation stage with GWAS data for MY, PP, and FP uncovered cell type‐specific enrichment of trait‐associated variants, identifying seven single‐nucleotide polymorphisms (SNPs) overlapping accessible chromatin regions in LASP4 and Lactocytes. Pseudotime analysis demonstrated differentiation trajectories of LASP4 toward Lactocytes and LHS cells. Combined snRNA‐seq, snATAC‐seq, and cell‐cell communication analyses further revealed lineage heterogeneity and functional diversification of Lactocytes and LHS cells during lactation compared with the dry stage. We also identified key transcription factors (TFs) and signaling pathways governing stage‐specific MEC differentiation. Cross‐species comparisons highlighted both conserved and divergent features of MECs across mammals, providing molecular insights into species‐specific milk composition. Altogether, these findings demonstrate that stage‐specific cellular interaction networks and transcriptional programs synergistically shape the spatial‐temporal precision of epithelial cell differentiation during the lactation cycle, as well as the resource will contribute to the development of the Farm animal Genotype‐Tissue Expression (FarmGTEx) project [27].

## 2 Results

### 2.1 snRNA‐seq and snATAC‐seq Atlases of the Dairy Goat Mammary Gland at the Dry and Lactation Stages

**FIGURE 1:**
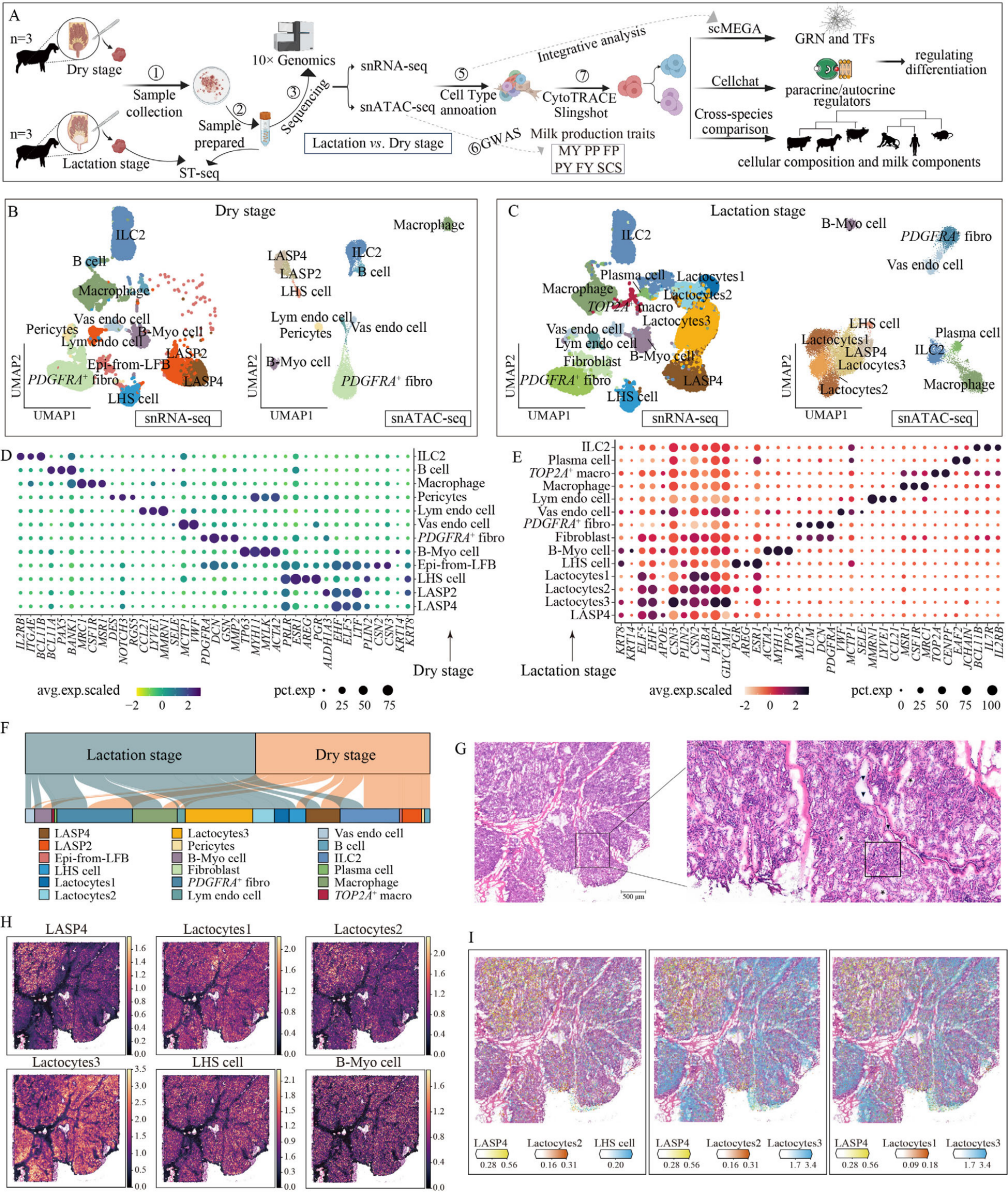
Major mammary epithelial, stromal and immune cell types identified by snRNA‐seq and snATAC‐seq. (A) A workflow of this study. Each arrow represents each method. (B,C) UMAPs of snRNA‐seq and snATAC‐seq. Mammary cells are colored by cell types at the dry and lactation stages, respectively. Different colors represent different cell types. (D,E) Dot plots of cell type‐specific markers for each cell type as identified by multi‐pairwise differential gene expression analysis of snRNA‐seq data at the dry and lactation stages, respectively. (F) The proportions of all annotated cell types at the dry and lactation stages. Color is identical to that in (B, D). Different colors represent different cell types, and the connecting lines indicate the correspondence between stages and different cell types. (G) Representative H&E staining of lactating mammary gland tissue showing well‐defined lobular and alveolar structures. A representative lobular structure is highlighted by a black box (right panel). Mammary ducts and alveolar structures are indicated by triangles and asterisks, respectively. (H) Cell2Location mapping of lactating goat mammary gland tissue sections reveals spatial alignment of annotated cell types with their expected anatomical structures. (I) Co‐localization analysis of distinct epithelial cell subtypes. Vas endo cells, Vascular endothelial cells; Lym endo cells, lymphatic endothelial cells; *PDGFRA* ^+^ fibro, *PDGFRA* ^+^ fibroblast; *TOP2A* ^+^ macro, *TOP2A* ^+^ macrophage; MY, milk yield; PP, protein percentage; FP, fat percentage; PY, protein yield; FY, fat yield; SCS, somatic cell score.

The lactation process is accompanied by substantial remodeling of MEC composition and gene expression patterns, while other structural cell types likely contribute to maintaining microenvironmental homeostasis and regulating cell turnover during this physiological transition [1, 28, 29]. To characterize the dynamic changes of MECs in dairy goats throughout the lactation cycle, we generated both snRNA‐seq and snATAC‐seq data from mammary glands at the dry and lactation stages, each with three biological replicates (Figure 1A). The snRNA‐seq yielded transcriptomic profiles of 27,915 and 36,284 nuclei for the dry and lactation stages, respectively (Table S1), while the snATAC‐seq provided chromatin accessibility profiles of 35,971 and 43,013 nuclei at the dry and lactation stages, respectively (Table S2). Based on canonical marker genes, 18 distinct cell types were annotated and grouped into 7 major cell classes based on their biological identities (Figure S1A–H), including alveolar cell class (expressing *EHF*, *ELF5* and *LTF*), such as LASP4 [12, 15, 30, 31, 32], luminal adaptive secretory precursor 2 (LASP2) and Epithelial cells from lobular fibroblast (Epi‐from‐LFB); three Lactocyte subtypes (Lactocytes1, Lactocytes2 and Lactocytes3) expressing *KRT8*, *CSN2*, *CSN3* and *LALBA*; LHS cells expressing *PGR*, *ESR1* and *AREG*; two B‐Myo cell types expressing *KRT14*, *TP63* and *MYH11*; two fibroblast types expressing *MMP2*, *DCN* and *APOD*; Vascular and lymphatic endothelial cells expressing *MMRN1*, *LYVE1* and *VWF*; Pericytes expressing *RGS*, *NOTCH3* and *DES*; and immune cells including ILC2, B cells, plasma cells, macrophages and *TOP2A* ^+^ macrophages. We also regressed out cell proliferation effects and found that, apart from *TOP2A* ^+^ macrophages, which indeed exhibited proliferative features, removing cell cycle signals did not substantially affect clustering or marker gene identification in other cell types (Figure S1I). In total, 12 and 14 cell types were identified at the dry and lactation stages, respectively (Figure 1B–E and Figure S1J), and the relative proportions of these cell types varied across stages (Figure 1F and Table S3). Except for Epi‐from‐LFB and *TOP2A* ^+^ macrophages, which were only detected in snRNA‐seq data, all other cell types were annotated in both snRNA‐seq and snATAC‐seq datasets, although discrepancies arose due to technical and thresholding differences between modalities [33].

Among the eight shared cell types, LASP4 exhibited high expression of genes related to milk synthesis and progenitor function, such as *PAEP*, *LTF*, *FASN*, *XDH*, *EHF*, and *ELF5* involved in biosynthesis of the milk components [34, 35, 36], epithelial differentiation [37, 38], and alveolar progenitor differentiation [39, 40]. Additional genes are associated with progenitor maintenance [41] and differentiation [42, 43], suggesting that LASP4 may have differentiation potential. Notably, 870 genes upregulated in lactation‐stage LASP4 were enriched in gene ontology (GO) terms related to lipid and protein biosynthesis, consistent with their known role in preparing for lactation initiation [12]. For LHS cells, 455 genes responded to the lactation‐promoting hormones prolactin, estrogen, and progesterone [44], were significantly upregulated at the lactation stage. Conversely, genes upregulated at the dry stage are known to mediate mammary gland involution and stromal remodeling in response to glucocorticoids and progesterone [45]. Additionally, B‐Myo cells at the lactation stage displayed 710 significantly upregulated genes involved in lipid particle disassembly, cellular homeostasis, and regulation of insulin‐like growth factor receptor signaling (Figure S2A and Table S4). Differential gene expression analysis in other cell types also revealed lactation‐enhanced functional specialization, including epithelial morphology maintenance, functional regulation and chemokine production, as well as increased stromal cell activity in mammary duct branching and epithelial polarity establishment (Figure S2B–D and Table S4).

For other present cell types, we found that LASP2, Epi‐from‐LFB, Pericytes and B cells were unique to the dry stage, while Lactocytes1, Lactocytes2, Lactocytes3, Fibroblasts, Plasma cells and *TOP2A* ^+^ macrophages were specific to the lactation stage. Further functional analyses of GO and Gene set enrichment analysis (GSEA) revealed distinct specializations among lactocyte subtypes. Lactocytes1 was enriched in metabolic signaling, translation, and amino acid metabolism, indicating a role in protein synthesis. In contrast, Lactocytes2 was associated with cell polarity and growth regulation, and showed strong enrichment in lipid metabolic and biosynthetic pathways, suggesting a primary role in lipid synthesis. Lactocytes3 was uniquely enriched in pathways related to cell‐cell junction organization and morphogenesis, particularly TGF‐β signaling (Figure S2E,F and Tables S5 and S6). Together, our snRNA‐seq and snATAC‐seq analyses revealed pronounced heterogeneity and stage‐specific specialization of MECs in dairy goats. During lactation, LASP4 retain differentiation potential, while lactocytes exhibit complex signaling and functional diversity. Immune and stromal cells also strengthened their supportive roles in maintaining epithelial integrity and mammary function.

To characterize the spatial distribution of lactation‐associated epithelial cell types, Hematoxylin and Eosin (H&E) staining of lactating mammary tissue revealed well‐defined lobular structures, ducts, and densely arranged alveolar (Figure 1G). Spatial transcriptomic analysis was then conducted to further investigate the molecular landscape of these epithelial compartments. At bin50 resolution, a total of 35,404 spots containing both gene expression profiles and positional information were obtained. We next applied Cell2location for spatial deconvolution, mapping snRNA‐seq defined cell types onto the mammary tissue sections, which successfully localized 14 distinct cell types to specific spatial regions. LASP4, Lactocytes1, Lactocytes2, Lactocytes3 and LHS cells were broadly distributed within mammary lobules. Notably, LASP4 and Lactocytes2 were enriched in specific lobular regions, whereas Lactocytes1 and Lactocytes3 were relatively concentrated in other areas (Figure 1H and Figure S3A,B). Spatial co‐localization analysis further revealed strong overlap of LASP4 with Lactocytes2 and LHS cells, along with regional co‐localization with multiple lactocyte subtypes (Figure 1I). Immunofluorescence (IF) staining additionally confirmed the presence of Lactocytes1, Lactocytes2, Lactocytes3, LHS cells and LASP4 in the lactating mammary gland tissue (Figure S3C). Overall, lactation‐stage MECs show enhanced transcriptional regulation, signaling activity and functional refinement, underscoring their dynamic plasticity in the lactation cycle.

### 2.2 Enrichment of Lactation‐Associated Variants and TF Motifs in Mammary Cell‐Type‐Specific Chromatin Accessibility

**FIGURE 2:**
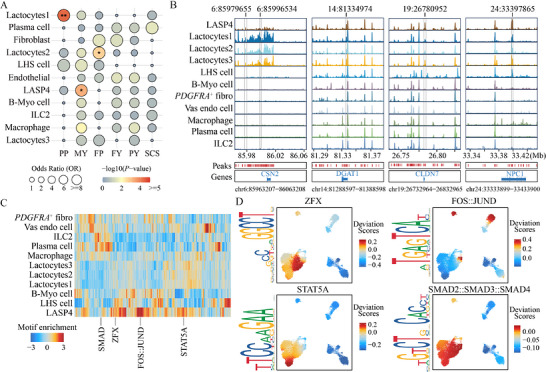
Enrichment of GWAS variants and TF motifs for lactation traits across mammary cell types. (A) Enrichment analysis of lactation‐associated SNPs in cell type‐specific chromatin accessibility regions. (B) Representative variants overlapped with open chromatin regions in Lactocytes and LASP4 at the *CSN2*, *DGAT1*, *CLDN7*, and *NPC1* loci. (C) Enrichment analysis of TF motifs in cell type‐specific accessible chromatin regions. The value of motif enrichment is a normalized measure of motif accessibility relative to background peaks. Red indicates higher motif enrichment within the accessible regions of the corresponding cell type. (D) TF motifs enriched in the cell type‐specific accessible chromatin regions of Lactocytes and LASP4. Vas endo cells, Vascular endothelial cells; Lym endo cells, lymphatic endothelial cells. *PDGFRA* ^+^ fibro, *PDGFRA* ^+^ fibroblast; MY, milk yield; PP, protein percentage; FP, fat percentage; PY, protein yield; FY, fat yield; SCS, somatic cell score.

Numerous SNP loci associated with milk production traits have been identified through GWAS in dairy goats. To gain deeper insight into the regulatory mechanisms underlying these loci, we evaluated the enrichment of 245 SNPs collected from published studies within cell type‐specific chromatin accessibility regions of the dairy goat mammary gland (Table S7) [23, 24, 25]. Fisher's exact test revealed that variants associated with MY were significantly enriched in LASP4 cell‐specific chromatin accessibility regions, variants associated with PP were significantly enriched in Lactocytes1‐specific regions, and variants associated with FP were significantly enriched in Lactocytes2‐specific regions. Although some trait‐associated SNPs did not reach statistical significance, they still exhibited strong associations with specific cell types, as reflected by high odds ratios (e.g., OR > 4), such as the associations of Lactocytes3 with MY, FY and FP, and of LHS cells with PP, FP and MY (Figure 2A). Furthermore, several previously reported SNPs associated with milk production traits also showed enrichment in chromatin accessibility regions of specific cell types. For example, variants associated with PP in the *CSN2* (chr6:85979655 and chr6:85996534) overlapped with specifically chromatin accessibility regions in three Lactocyte subtypes. Similarly, FP‐related variants in *DGAT1* (chr14:81334974) overlapped with a chromatin accessibility region in LASP4 and three Lactocyte subtypes. The precise mapping of these milk production trait‐associated variants, combined with their overlap to cell type‐specific chromatin accessibility, provides further evidence that these epithelial cell types may contribute to the corresponding milk production traits. Notably, in LASP4‐specific chromatin accessibility region, CLDN7 overlapped with the MY‐, FY‐, and PY‐associated SNP chr19:26780952, while NPC1 overlapped with the MY‐associated SNP chr24:33397865 (Figure 2B). Both genes have been reported as molecular markers during lactation and are involved in the maintenance of cell polarity and cholesterol transport, respectively [46, 47].

In addition to linking complex traits to SNP loci at the single‐cell level, we further associated them with TF motif enrichment. We hypothesized that integrative analysis could reveal how TFs regulate trait‐associated chromatin accessibility. Motif enrichment analysis on the cell type‐specific chromatin accessibility region for each cell type was performed and showed that motifs enriched in mammary‐specific populations aligned with well‐established mammary TFs. For example, motifs corresponding to lactation‐initiating TFs such as members of the STAT family and ZBTB7B were highly enriched in the chromatin accessibility region of LASP4 and Lactocyte subtypes. LHS cell was enriched in the *ZFX* motif (Table S8), which is involved in sex chromosome regulation and hormone responsiveness [48]. Importantly, LASP4‐specific accessible regions were enriched with the SMAD family and FOS::JUND. The SMAD family has been implicated in maintaining mammary epithelial progenitor identity and promoting milk fat synthesis [49, 50, 51, 52], while FOS::JUND has also been shown to contribute to the regulation of mammary stem cell pluripotency and lineage differentiation during development [53]. SMAD motif enrichment was also observed in other epithelial subtypes (Figure 2C,D), suggesting that these TFs may influence lactation‐related gene expression and epithelial differentiation across diverse cellular states.

Together, by analyzing the overlap between trait‐associated SNPs and chromatin accessibility regions in mammary‐specific cell types, we established links among non‐coding variation, cell types and milk production traits in the goat mammary gland. Our findings highlight the preferential enrichment of SNPs associated with MY, PP and FP in the chromatin accessibility region of LASP4 and three distinct Lactocyte subtypes, and reveal unique transcriptional regulatory networks within these subpopulations. In particular, the identification of key non‐coding variants and the enrichment of SMAD2 and FOS::JUND motifs in LASP4‐specific accessible regions suggest that these variants and TFs may modulate lactation phenotypes by influencing cell differentiation and the maintenance of epithelial polarity.

### 2.3 Dynamic Heterogeneity of Epithelial Cells at Different Lactation Stages

**FIGURE 3:**
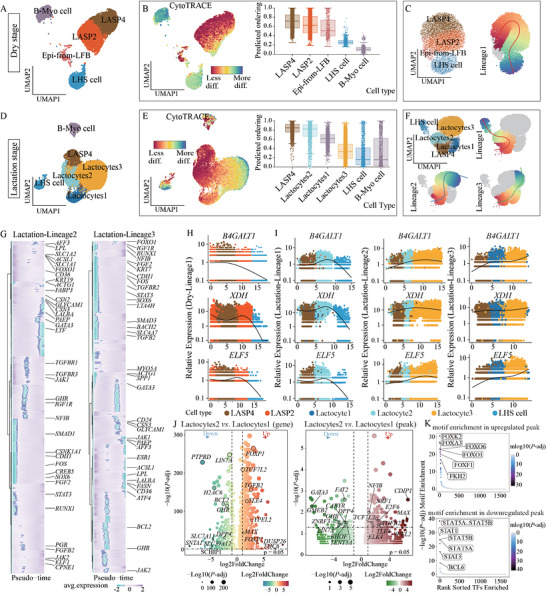
The heterogeneity and diverse differentiation trajectories of mammary epithelial cells (MECs) at the dry and lactation stages. (A,D) UMAP of subcellular clustering of MECs at the dry and lactation stages, with different colors representing distinct MEC populations, respectively. (B,E) CytoTRACE score plot for stemness analysis of MECs at the dry and lactation stages, respectively. Dark‐green indicates lower scores (low stemness), while dark‐red indicates higher scores (high stemness). The bar chart quantifies the scores from the UMAP, with colors representing different cell types, consistent with the colors in (A, D). (C,F) Demonstration of the distribution of slingshot‐predicted MEC differentiation trajectories by UMAP plots. Each spectrum was plotted according to the pseudotime value to infer the differentiation trajectory. The color from red to blue indicates the pseudotime from naive to mature, and the grey part of the cells represents no belonging to the lineage. The UMAP plot displays the distribution of cell types along a differentiation trajectory at the dry stage. Dry‐Lineage1 from naive to mature: progresses from LASP4 to LASP2 and then to LHS cell in (C). There are three differentiation trajectories from native to mature at the lactation stage. Lactation‐Lineage1: progresses from LASP4 to Lactocytes2 and then to LHS cell; Lactation‐Lineage2: progresses from LASP4 to Lactocytes2 and then to Lactocytes3; Lactation‐Lineage3: progresses from LASP4 to Lactocytes1 and then to Lactocytes3 in (F). (G) Heatmap of genes exhibiting pseudotime‐dependent changes along the differentiation trajectory of Lactation‐Lineage2 and ‐Lineage3. (H–I) Three genes exhibit pseudotime‐dependent changes along the differentiation trajectory of dry (H) and lactation (I) stages. (J) The left and right figures represent the differentially expressed genes and differential peaks of Lactocytes2 compared to Lactocytes1, respectively. (K) Motif enrichment analysis of differential peaks in Lactocytes2 compared to Lactocytes1.

Among mammary cell types, epithelial cells serve as the primary effectors of lactation, orchestrating milk synthesis and secretion through highly specialized structures and functions [54]. To explore the phenotypic and functional transitions of MECs during lactation and compare them with those in the dry stage, we evaluated the stage‐specific differentiation potentials and subsequently reconstructed lineage trajectories. The results showed that LASP4 exhibited the highest stemness scores at both stages (Figure 3A,B and Figure 3D,E), indicating their differentiation potential throughout lactation. Trajectory analysis confirmed this observation: LASP4 differentiated from LASP4 to LASP2 and then to LHS cell (Dry‐Lineage1) at the dry stage (Figure 3C). At the lactation stage, LASP4 followed three differentiation trajectories: the progresses from LASP4 to Lactocytes2 and then to LHS cell (Lactation‐Lineage1); the progresses from LASP4 to Lactocytes2 and then to Lactocytes3 (Lactation‐Lineage2); and the progresses from LASP4 to Lactocytes1 and then to Lactocytes3 (Lactation‐Lineage3) (Figure 3F). These findings indicate that phenotypically distinct LHS cells at different stages originate from LASP4, which also possess the potential to differentiate into different Lactocyte subtypes. This highlights the remarkable plasticity of LASP4 and the critical role of LHS cells under different lactational states.

To validate the reconstructed differentiation trajectories, we analyzed genes dynamically expressed along the differentiation trajectories (Figure 3G and Figure S4A,B). We found that hormone receptor genes *PGR*, *ESR1*, and *PRLR* were continuously upregulated along Dry‐ and Lactation‐Lineage1, while remaining unchanged in Lactation‐Lineage2/3 (Figure S4C). In contrast, lactation‐related genes (e.g., *CSN2*, *CSN3*, *GLYCAM1*) were consistently upregulated in Lactation‐Lineage2/3, but not in Lactation‐Lineage1 (Figure S4D). These expression patterns are consistent with the functional transitions of cells at different lactation stages. Other key genes, involved in milk synthesis and alveolar development, such as *XDH* [37, 55], *ELF5* [12, 39], *B4GALT1* [56], and *ACACA* [55, 57] showed transiently upregulated in Dry‐ and Lactation‐Lineage1 but sustained expression in Lactation‐Lineage2/3 (Figure 3H,I). The lactation‐related gene, known to be involved in milk lactose and fat synthesis, was also consistently upregulated in Lactation‐Lineage2/3 (Figure S4E). Stemness‐related genes, including *MAST4* [58, 59] and *CCNY* [60], were differentially expressed in Lineage1 and Lineage2/3, suggesting their roles as critical regulators in directing progenitor cells toward specific lineages (Figure S4E). These patterns are consistent with the functions of genes highly expressed in LASP4, further underscoring their capacity as progenitor cells with differentiation potential, giving rise to functionally distinct epithelial subtypes through various trajectories that collectively contribute to lactation.

To further explore differences between Lactation‐Lineage2 and ‐Lineage3, we compared gene expression profiles and chromatin accessibility in the two intermediate cell types, Lactocytes1 and Lactocytes2. We identified 834 differentially upregulated differential expression gene (DEGs) in Lactocytes2, and 169 genes associated with regions of increased chromatin accessibility, of which 42 were shared, suggesting their transcriptional upregulation may be driven by enhanced chromatin accessibility. Among them, *TLE4* has previously been reported to be involved in the synthesis of milk proteins [61]. In addition, 328 DEGs were upregulated in Lactocytes1, and 53 chromatin accessibility regions were annotated to upregulated genes, of which 9 genes were shared, such as *GHR* that is known to participate in milk fat and lactose synthesis [62]. These functional genes are likely to facilitate the differentiation of LASP4 toward Lactocytes3 through two distinct intermediate subtypes (Figure 3J). We performed motif enrichment analysis on differentially chromatin accessibility regions upregulated in Lactocytes2 and Lactocytes1. In Lactocytes2, 108 enriched TF motifs were identified, with top‐ranked TFs including *FKH2*, *FOXO1* and *FOXO6*, which are known to promote milk fat synthesis in dairy goats [63, 64]. In contrast, Lactocytes1 revealed 27 enriched TF motifs, with STAT5 being the most prominent (Figure 3K). *STAT5* and its variants (*STAT5A*/*B*) form homodimers that translocate into the nucleus to activate milk protein synthesis genes [65, 66]. These results confirm that Lactocytes1 is specialized for milk protein synthesis, while Lactocytes2 is associated with milk fat synthesis. Their distinct gene expression and chromatin accessibility patterns drive this functional divergence, ensuring balanced milk composition through bifurcated LASP4 differentiation.

### 2.4 Identification of TFs Regulating the Differentiation of Milk‐Secreting Cells

**FIGURE 4:**
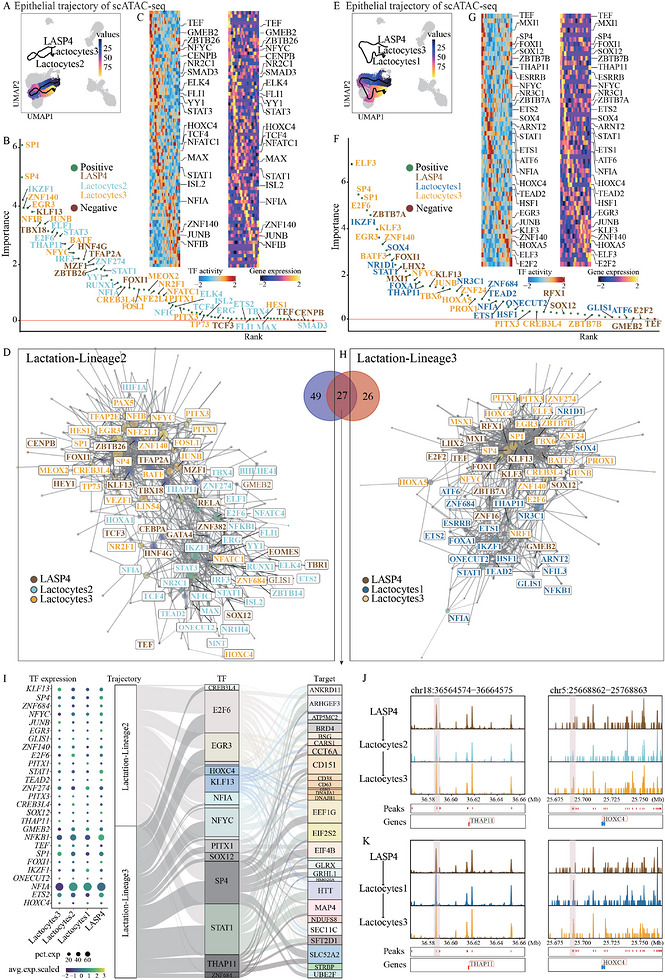
Identification of TFs regulating the differentiation of milk‐secreting cells and construction of gene regulatory networks (GRN). (A,E) Pseudotime analysis of Lactocytes differentiation trajectories for Lactation‐Lineage2/3 through snATAC‐seq data based on the trajectories of snRNA‐seq using ArchR, respectively. (B,F) Importance ranking of TFs in cell types along the differentiation trajectories of Lactation‐Lineage2/3, with font colors indicating different cell types. (C,G) Heatmap showing TF binding activity and gene expression along the differentiation trajectories of Lactation‐Lineage2/3. (D,H) GRNs for Lactation‐Lineage2/3, where each node represents a TF or a gene. Venn diagram illustrates the overlap of TFs within the GRNs associated with Lactation‐Lineage2/3. The arrows pointing to Figures I and J indicate that the analyses were conducted using these overlapping TFs. (I) Expression levels and target genes of shared TFs in cell types along Lactation‐Lineage2/3. The heatmap displays the expression levels of overlapping TFs in cell types along Lactation‐Lineage2/3, while the Sankey diagram illustrates the target genes of these shared TFs. (J–K) The accessibility of peaks for the shared TFs (THAP11 and HOXC4) across cell types included in Lactation‐Lineage2/3.

LASP4 exhibits greater plasticity at the lactation stage compared to the dry stage. Their differentiation into the Lactocytes lineage is essential for secretory activation, driven by lineage‐specific TF‐mediated networks and chromatin remodeling that coordinately activate lactogenic genes. To understand the roles of TFs in these differentiation trajectories and the underlying molecular mechanisms governing these complex processes, we integrated snATAC‐seq and snRNA‐seq data to analyze chromatin accessibility and constructed GRNs for each trajectory. First, we found that the MEC differentiation trajectories inferred from snATAC‐seq data were consistent with those obtained from snRNA‐seq data, revealing two paths: Lactation‐Lineage2 and Lactation‐Lineage3 (Figure 4A,E). Subsequent TF importance analysis identified 29 TFs with high regulatory potential (importance score >1) in Lactation‐Lineage2, with SP1, SP4, and THAP11 showing the highest regulatory potential (Figure 4B and Table S9). Notably, SP1 is known to regulate lipid metabolism genes, such as *PPARγ* and *LXRα*, promoting milk fat synthesis [67]. In Lactation‐Lineage3, 24 TFs showed high importance scores (importance score >1), with ELF3, SP4, KLF3 and THAP11 standing out as key factors (Figure 4F and Table S9). ELF3, in particular, has been identified to directly associate with milk protein synthesis through GWAS [68]. Integrative analysis further revealed that both the expression levels and chromatin accessibility of these TFs gradually increased along the differentiation trajectories (Figure 4C,G).

Next, we integrated snRNA‐seq and snATAC‐seq data to construct GRNs and identified core TFs involved in the two differentiation lineages (Figure 4D,H). After excluding TFs associated with LHS cells, we identified 34 and 14 lineage‐specific TFs in the GRNs of Lactation‐Lineage2 and ‐Lineage3, respectively. In Lactation‐Lineage2, 12 TFs exhibited a continuous increase in expression along the trajectory, peaking in Lactocytes3. Among them, *CEBPA* [69] and *NFE2L1* [70] have previously been implicated in the synthesis of milk components and maintenance of lactational homeostasis. In Lactation‐Lineage3, ATF6, BATF3, ELF3 and ZNF24 showed similar upregulation dynamics, showing their highest expression levels in Lactocytes3. Notably, both ATF6 and ELF3 have been reported to play essential roles in milk protein synthesis [71], suggesting their potential regulatory roles in driving Lactation‐Lineage3 differentiation.

In addition, we identified 17 TFs shared between the two lineages, with *THAP11* and *HOXC4* showing continuous upregulation along both differentiation trajectories and remaining highly expressed in Lactocytes3, suggesting key roles in LASP4 differentiation into Lactocyte subtypes. Chromatin accessibility analysis further revealed a progressive increase in THAP11 accessibility from LASP4 to Lactocytes3, whereas HOXC4 exhibited a different pattern (Figure 4K). *HOXC4*, known to directly regulate CSN3 expression and is typically highly expressed in fully differentiated cells, where it plays a direct role in promoting terminal differentiation [72, 73]. Conversely, *THAP11* is enriched in progenitor populations with high plasticity [74]. It binds to ultra‐conserved promoter elements in developmental genes and modulates transcription across diverse biological contexts [75]. Through interactions with the transcriptional co‐regulator HCF‐1 and chromatin‐modifying proteins, *THAP11* also participates in pluripotency regulation via non‐canonical pathways [74, 76]. Its conserved THAP domain, a zinc finger DNA‐binding motif, facilitates broad chromatin regulatory functions [74]. These findings suggest *THAP11* as a key regulator of LASP4 lineage commitment and Lactocytes differentiation by modulating chromatin accessibility (Figure 4J).

Overall, our findings demonstrate that secretory‐competent Lactocytes differentiate along two distinct trajectories in the lactating mammary glands: Lactation‐Lineage2 and ‐Lineage3, with each characterized by specialized functional roles. Despite their functional divergence, both lineages act in concert to sustain the continuous synthesis of milk components. Importantly, *THAP11* emerged as a shared regulatory hub across both lineages, essential for maintaining the Lactocytes differentiation program. These results not only elucidate the transcriptional architecture underlying lactational specialization but also highlight potential molecular targets for future functional studies.

### 2.5 Identification of TFs Regulating the Differentiation of Luminal Hormone‐Sensing Cell

**FIGURE 5:**
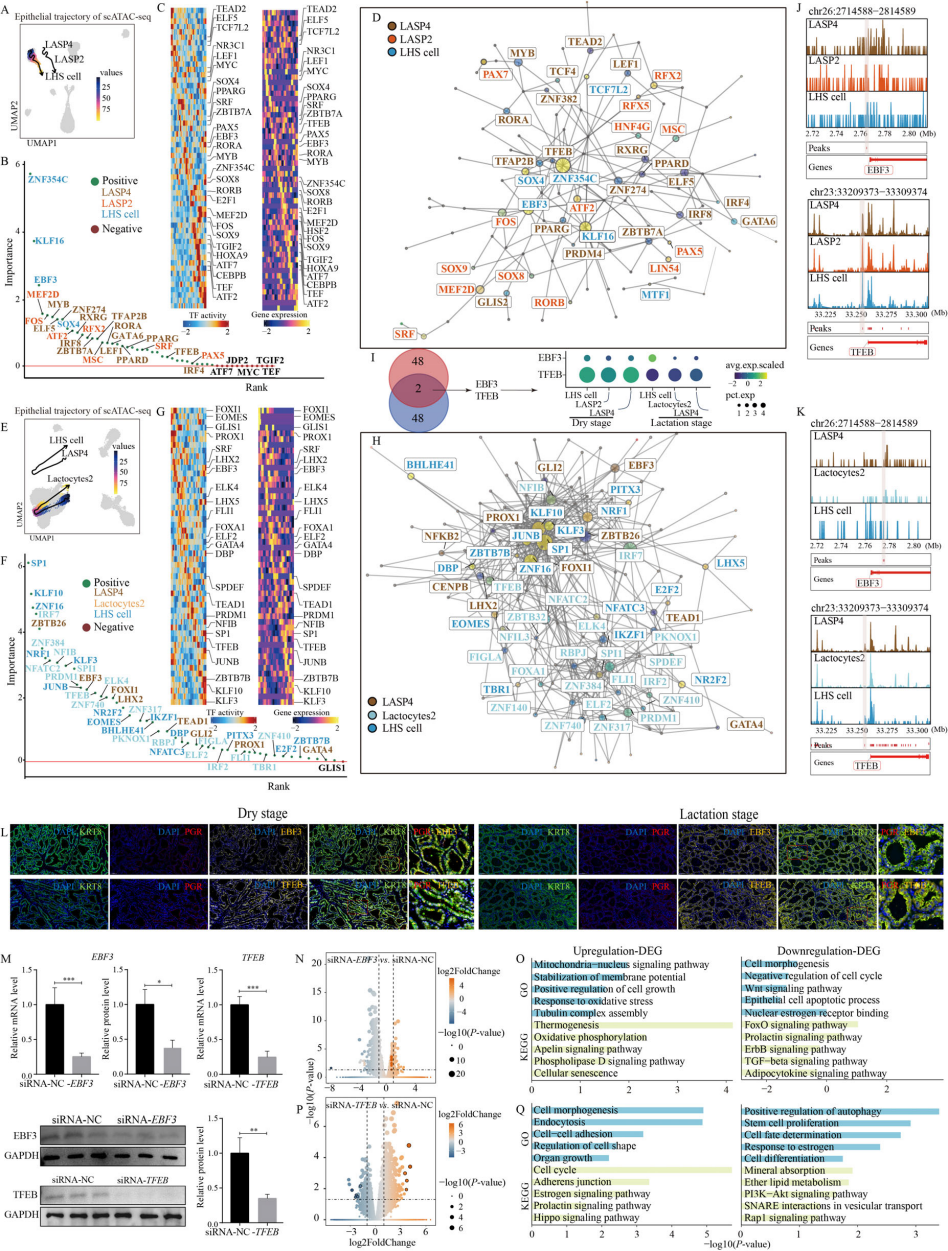
Identification of TFs regulating the differentiation of luminal hormone‐sensing cell and construction of gene regulatory networks (GRN). (A,E) Pseudotime analysis of LHS cell differentiation for Dry‐ and Lactation‐Lineage1 through snATAC‐seq data based on the trajectories of snRNA‐seq using ArchR, respectively. (B,F) Importance ranking of TFs in cell types along the differentiation trajectories of Dry‐ and Lactation‐Lineage1, with font colors indicating different cell types. (C,G) Heatmap showing TF binding activity and gene expression along the differentiation trajectories of Dry‐ and Lactation‐Lineage1. (D,H) GRN analysis of corresponding cell types along the differentiation trajectories in Dry‐ and Lactation‐Lineage1, where each node represents a TF or a gene. (I) The Venn diagram illustrates the overlap of TFs within the GRNs and their expression levels in Dry‐ and Lactation‐Lineage1. (J–K) The accessibility of peaks for the overlapping TFs (EBF3 and TFEB) across cell types included in Dry‐ and Lactation‐Lineage1. (L) Immunofluorescence co‐localization of the core TFs EBF3 and TFEB with marker proteins of distinct cell types along the Dry‐ and Lactation‐Lineage1 differentiation trajectories. Immunofluorescence staining revealed that, along the Dry‐Lineage1 trajectory, EBF3 and TFEB were co‐localized with marker proteins of LHS cell (*PGR* and *KRT8*), respectively. (M) Knockdown efficiency of *EBF3* and *TFEB* in GMECs was assessed at the mRNA and protein levels by RT‐qPCR and Western blotting, respectively. (N, P) Volcano plots visualize DEGs in GMECs after knockdown of (N) *EBF3* (siRNA‐*EBF3* vs. NC) and (P) *TFEB* (siRNA‐*TFEB* vs. NC). (O,Q) GO and KEGG pathway enrichment analyses of upregulated and downregulated DEGs from (O) siRNA‐*EBF3* and (Q) siRNA‐*TFEB* groups. DEGs, differentially expressed genes. siRNA‐NC, negative control; siRNA‐*EBF3*, *EBF3* knockdown group; siRNA‐*TFEB*, *TFEB* knockdown group; Data are presented as mean ± SD; ^*^ *p* < 0.05, ^**^ *p* < 0.01, ^***^ *p* <0.001.

Although LHS cells exhibit less complex differentiation trajectories compared to Lactocytes, they are indispensable for mammary tissue remodeling and lactational performance at the dry and lactation stages [4]. To elucidate how LASP4 differentiate into functionally distinct LHS cells, we analyzed gene regulatory programs using snATAC‐seq data from the mammary gland of a dairy goat. Pseudotime trajectory analysis of snATAC‐seq data revealed two stage‐specific differentiation trajectories from LASP4 to LHS cells, termed Dry‐Lineage1 and Lactation‐Lineage1 (Figure 5A,E). Next, we integrated snRNA‐seq and snATAC‐seq data to construct GRNs for these differentiation trajectories, and evaluated TF importance scores. Comparative analysis of the GRNs from the two stages identified 48 unique TFs specifically regulating the distinct differentiation pathways (Figure 5D,H). Eight TFs showed high importance scores (importance score >1) in Dry‐Lineage1 (Figure 5B and Table S9), with five TFs (*KLF16*, *MEF2D*, *MYB*, *ZNF274* and *ZNF354C*) exhibiting concordant expression and chromatin accessibility patterns, indicating high activity from LASP4 to LHS cell differentiation (Figure 5C). These top TFs may participate in LHS cell functional specification and terminal differentiation. For instance, MYB has been reported as a direct target of estrogen/ER signaling [77], and *MEF2D* responds to steroid hormone stimulation, which may promote epithelial progenitor cell differentiation [78]. Meanwhile, Lactation‐Lineage1 involved 17 high‐importance TFs (importance score >1) (Figure 5F and Table S9), with *SP1*, *KLF10* and *ZNF15* showing the highest scores. Additionally, *IRF7*, *JUNB*, *NRF1*, *ZBTB26* and *ZNF16* were progressively upregulated, peaking in LHS cells and accompanied by increasing chromatin accessibility (Figure 5G).

Among the TFs involved in the differentiation trajectories, *EBF3* and *TFEB* emerged as key regulators shared by both lineages. To further validate their regulatory roles during mammary gland development, we examined their endogenous expression patterns and biological functions in the goat mammary gland. IF staining was first performed to assess the expression and subcellular localization of EBF3 and TFEB in mammary tissues at the dry and lactation stages. Co‐localization analyses revealed that at the dry stage, both TFs were predominantly expressed in LASP2, LASP4 and LHS cells, whereas at the lactation stage, their expression was mainly detected in LASP4, Lactocytes2 and LHS cells (Figure 5L and Figure S5A).

To further investigate their endogenous functions, we individually silenced *EBF3* and *TFEB* expression in goat MECs (GMECs) using specific siRNAs. RT‐qPCR and Western blot analyses confirmed significant reductions in both mRNA and protein levels, indicating efficient knockdown of their endogenous *EBF3* and *TFEB* (Figure 5M and Figure S5B). Subsequently, bulk RNA‐seq (Table S10) was performed on GMECs transfected with siRNA targeting *EBF3* or *TFEB*, along with a negative control (NC), to systematically evaluate the biological consequences of their depletion. Compared with the NC group, the siRNA‐*EBF3* group exhibited 1163 DEGs, including 359 upregulated and 804 downregulated DEGs (Figure 5N and Table S11). GO and KEGG analyses showed that EBF3 is essential for maintaining the differentiated state of MECs and hormone‐responsive signaling programs, while suppressing stress‐related and compensatory growth pathways, thereby supporting mammary tissue remodeling and the stability of lactational function (Figure 5O and Table S11). For TFEB, a total of 1,165 DEGs were identified in the siRNA‐*TFEB* group compared with the NC group, including 891 upregulated and 274 downregulated genes (Figure 5P and Table S11). GO and KEGG analyses of the DEGs indicated that TFEB primarily regulates autophagy, cell fate determination, and differentiation‐related transcriptional programs, thereby promoting the transition of MECs from a proliferative to a differentiated state and playing an essential role in mammary gland renewal and remodeling at the dry stage (Figure 5Q and Table S11).

Taken together, these findings validate that both Dry‐ and Lactation‐Lineage 1 originate from LASP4 and differentiate into LHS cells, yet they are regulated by stage‐specific GRNs, where TFs at the dry stage mainly sustain basal cellular functions, while those at the lactation stage primarily regulate metabolic and immune programs. Among them, *EBF3* and *TFEB* act as shared regulators across both trajectories, serving as a conserved regulatory hub for LHS cell differentiation independent of physiological stage.

### 2.6 Cell Communication is a Potential Driver of LASP4 Differentiation

**FIGURE 6:**
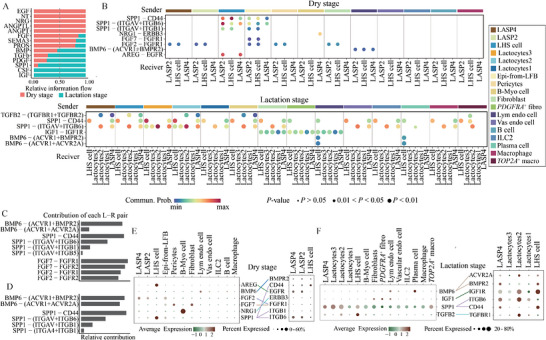
Cell–cell communication between other cell types and epithelial cells at different stages. (A) All the significant signaling pathways were ranked based on their differences of overall information flow within the inferred networks between dry and lactation stage. The overall information flow of a signaling network is calculated by summarizing all the communication probabilities in that network. The top signaling pathways colored by red are more enriched at the dry stage, and the bottom ones colored by green are more enriched at the lactation stage. (B) Comparison of the significant ligand‐receptor pairs that contribute to the signaling from stromal and immune cells to MECs between dry and lactation stage. Dot color reflects communication probabilities and dot size represents computed *p*‐values. Empty space means that the communication probability is zero. *p*‐values are computed from one‐sided permutation test. (C,D) The signaling pathways of dry and lactation stage were ranked based on their pairwise Euclidean distance in the shared two‐dimensional manifold. Larger distance implies larger difference. (E,F) Expression of ligand genes in sender cell types and of receptor genes in MECs within key signaling pathways at the dry and lactation stages. Vas endo cells, Vascular endothelial cells; Lym endo cells, lymphatic endothelial cells; *PDGFRA* ^+^ fibro, *PDGFRA* ^+^ fibroblast; *TOP2A* ^+^ macro, *TOP2A* ^+^ macrophage.

To investigate potential ligand‐receptor (L‐R) pairs involved in LASP4 differentiation, we employed CellChat to infer intercellular communication networks at both dry and lactation stages. We identified 12 and 9 signaling pathways at the dry and lactation stages, respectively, with seven pathways shared between stages (Figure 6A). For each signaling pathway, distinct cell types displayed unique sender‐receiver interaction patterns (Figure S6A and Table S12). Considering MECs as signal recipients, epithelial differentiation at the dry stage was mainly regulated by SPP1, FGF, NRG, BMP, and EGF signaling, primarily derived from stromal and epithelial cells (Figure 6B and Figure S6B–F). In contrast, at the lactation stage, SPP1, BMP, IGF and TGFβ pathways predominated, with all cell types contributing ligands (Figure 6B and Figure S6I–L). SPP1 and TGFβ signaling exhibited autocrine and paracrine interactions, while BMP and IGF signaling were exclusively paracrine. SPP1 was broadly produced by epithelial, stromal, and immune cells, suggesting a complex regulatory network (Figure S6F).

During LASP4‐to‐LHS differentiation, clear stage‐specific patterns were observed. At the dry stage, LHS cell secreted SPP1 and AREG, activating SPP1 and EGF signaling in LASP4 and LASP2 by binding to the ITGAV/ITGB6 complex and EGFR, respectively (Figure 6B,C), consistent with the high expression of SPP1 and AREG in LHS cell and elevated ITGB6 and EGFR levels in LASP4 and LASP2 (Figure 6E). LHS cell also secreted SPP1 ligands acting on CD44 in LASP2, although CD44 expression was relatively low. In contrast, at the lactation stage, LHS cell maintained SPP1 signaling by secreting SPP1 ligands that bound to the ITGAV/ITGB6 complex, with ITGB6 highly expressed in LASP4 and Lactocytes2. These results suggest that SPP1 signaling via the SPP1‐(ITGAV/ITGB6) pair promotes LASP4 differentiation to LHS cell at both two stages. However, EGF signaling through the AREG‐EGFR pair we only observed at the dry stage, potentially contributing to the distinct differentiation trajectories between the two stages. Notably, previous studies have highlighted the role of AREG‐EGFR signaling in maintaining mammary ductal morphology [56].

To investigate the key autocrine/paracrine signals driving LASP4 differentiation toward Lactocytes, we analyzed cell‐cell communication between epithelial and all cell types. We found that LASP4, Lactocytes2, and Lactocytes3 secreted SPP1 ligands that activated ITGAV/ITGB6 receptor, thereby promoting SPP1 signaling and facilitating LASP4 differentiation toward Lactocytes3. In addition, plasma cells secreted high levels of BMP6 acted on BMPR2‐enriched LASP4 and ACVR2A‐expressing Lactocytes2, suggesting a role for BMP signaling in the transition from LASP4 to Lactocytes2 and Lactocytes3 (Figure 6E,F). Previous studies have well characterized the role of BMP signaling in mammary morphogenesis and progenitor cell differentiation [33]. Furthermore, fibroblasts and lymphatic endothelial cells mediated IGF signaling to Lactocytes2 via IGFR, while LHS cells activated TGFβ signaling via TGFB2 (Figure 6B,D,F), potentially promoting epithelial proliferation and extracellular matrix remodeling [79]. These findings highlight BMP, IGF, and TGFβ signaling as potential regulators of the Lactation‐Lineage2 differentiation trajectory. To further investigate the spatial distribution of these L‐R interactions, we analyzed their co‐expression patterns and predicted interaction strengths based on spatial transcriptomic data. The results showed that IGF1‐IGF1R, SPP1‐CD44/ITGB6, and TGFB2‐TGFBR1 were broadly distributed across the tissue and exhibited stronger interaction signals in regions enriched for LASP4 and Lactocytes2, consistent with the L‐R interactions identified from single‐cell analysis. In contrast, BMP6‐ACVR2A/BMPR2 showed co‐expression in some regions but did not display significant enrichment, likely due to its relatively low overall expression levels (Figure S7).

To further characterize global intercellular communication, we predicted interactions among epithelial, stromal, and immune cells at both two stages (Figure S8). B‐Myo cell mainly communicated through FGF signaling at the dry stage to SPP1 signaling during lactation. Stromal cells displayed stage‐dependent signaling shifts, predominantly using SEMA3 signaling at the dry stage and transitioning to SPP1 and PDGF signaling during lactation. Notably, PDGF signaling has been shown to facilitate stromal to Lactocytes transdifferention results also revealed stage‐specific secretory profiles in fibroblasts. Fibroblasts exhibited stage‐specific secretion patterns, producing FGF7 at the dry stage and switching to IGF1 during lactation, supporting epithelial differentiation and milk production. At the dry stage, LHS cells secreted AREG and SPP1 to activate EGF and SPP1 pathways, regulating ductal remodeling and alveolar formation. During lactation, LHS cell switched to TGFB2 and SPP1 secretion, engaging TGFβ and SPP1 signaling to suppress epithelial apoptosis, enhance milk protein expression, and potentially promote LASP4 differentiation into Lactocytes (Lactation‐Lineage2/3). Collectively, these stage‐specific TFs and autocrine/paracrine signals coordinate epithelial differentiation across the lactation cycle, ensuring efficient mammary gland remodeling and optimal milk production in dairy goats.

### 2.7 Conservation and Heterogeneity of Cell Types and Lactation Processes From Mammary Glands Across Six Species

**FIGURE 7:**
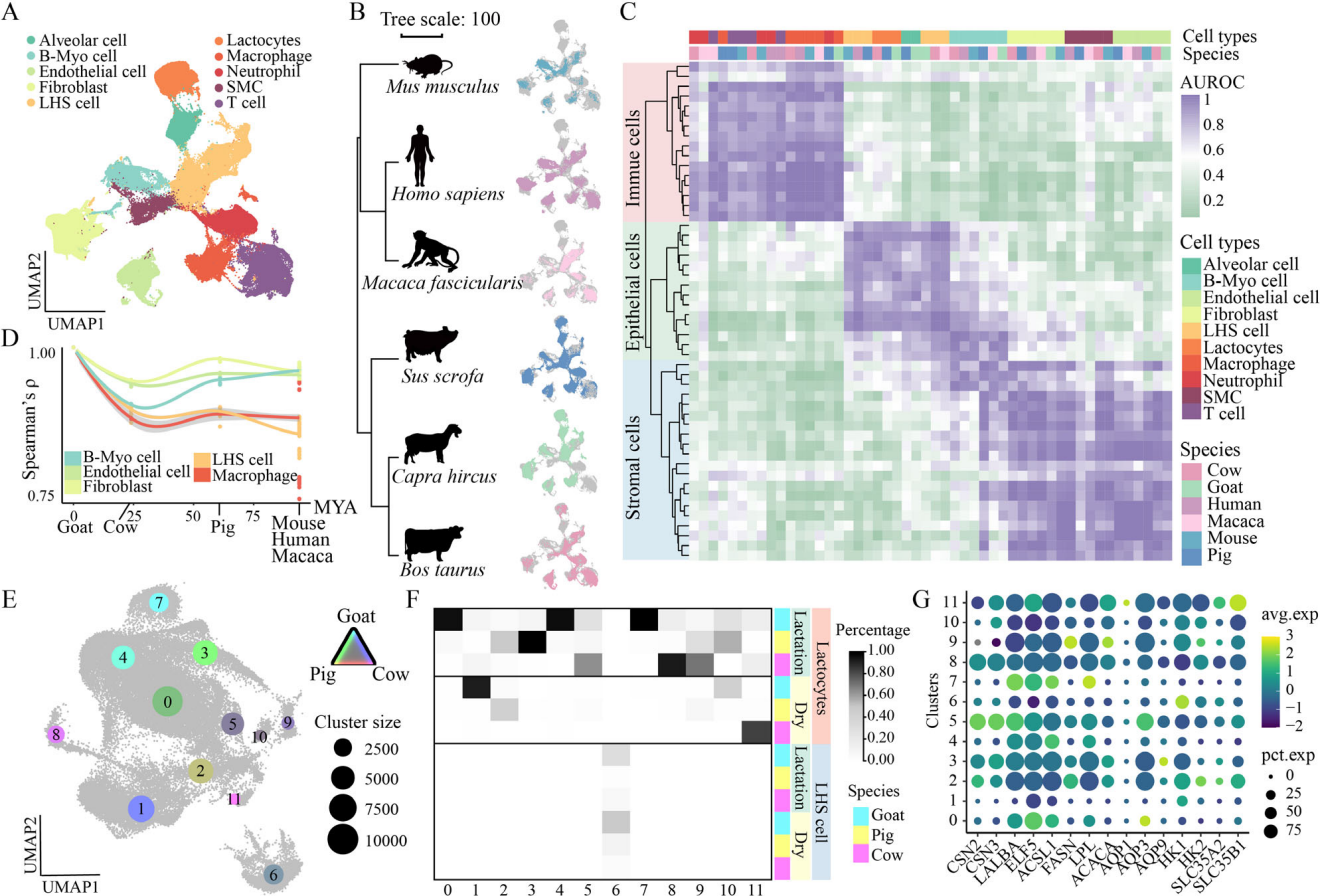
Cross‐species comparison of cell types and lactation processes from mammary glands across six species. (A) UMAP plot showing the integration of single‐cell transcriptomic data from mammary glands of dairy goats, cattle, pigs, macaques, humans and mice. (B) UMAP plots showing the distribution of mammary gland cells from each species. Different colors represent different species. (C) Hierarchical clustering of structural cells based on AUROC scores. Different colors of the rows above the heatmap represent different species or cell types, and the different colors in the heatmap represent the Spearman′s correlation coefficient calculated by AUROC scores. (D) Correlation analysis of gene expression profiles for five cell types shared among six species. (E) UMAP plot showing the integration of MECs from three species. Dot colors represent the species composition within each integrated cluster, and dot size corresponds to the number of cells. (F) The percentage of MECs in integrated clusters, and boxes are colored by specie mixture. (G) Expression levels of representative genes related to milk protein, lactose, and milk fat in integrated clusters.

The mammary gland of dairy goats exhibits distinct cell types and gene expression patterns that coordinate the initiation and maintenance of lactation. To investigate evolutionarily conserved cell types and transcriptional programs underlying mammalian lactation, as well as to identify goat‐specific molecular features, we generated snRNA‐seq data from mammary glands of cows, pigs, macaques, humans, and mice. Cross‐species comparisons were conducted using 1:1 orthologous genes shared across all six species (Table S13). Integration and cell type annotation identified ten distinct cell types categorized into three major classes: epithelial, immune and stromal cells. Among these, epithelial cells, including alveolar cells, Lactocytes, LHS cells and B‐Myo cells, were the predominant population across all species. Notably, Lactocytes were present in dairy goats, cows and pigs but absent in macaques, humans and mice, likely due to differences in lactation status at sampling. Immune cells included macrophages, neutrophils and T cells, while fibroblasts, smooth muscle cells (SMCs) and endothelial cells formed the stromal cell types (Figure 7A,B). Further cross‐species similarity analysis revealed that the three major cell classes formed distinct branches across the six species. Stromal cells, including fibroblasts, SMCs and endothelial cells, exhibited high interspecies conservation. Among epithelial cells, B‐Myo cell also displayed high interspecies similarity. In contrast, Lactocytes from dairy goats, cows and pigs clustered together, suggesting that their gene expression similarity is primarily driven by cell identity rather than species origin. Other epithelial subtypes, such as alveolar cells and LHS cells, as well as immune cell types, displayed greater variability and did not cluster strictly by cell type or species (Figure 7C).

To further examine evolutionary dynamics, we performed pseudo‐bulk analyses on five cell types, each represented by over 200 cells across all six species. These analyses assessed the evolutionary rates of mammary cell types. The results revealed that LHS cell exhibited the fastest evolutionary rates, while fibroblasts, B‐Myo cells, and endothelial cells evolved slower rates, consistent with their high cross‐species similarity. These findings suggest that the more conserved cell types are under stronger functional constraints (Figure 7D). In contrast, the accelerated evolution of epithelial cells, particularly LHS cells, likely reflects species‐specific adaptation to lactation demands, with transcriptional heterogeneity supporting specialized strategies for milk production.

Next, we compared Lactocytes and LHS cells from the lactating and non‐lactating stages across three species. Based on 1:1 homologous genes integration, we identified 12 distinct luminal cell subclusters through dimensionality reduction. We then examined the expression of representative genes associated with milk components in these cell populations. Among the integrated clusters, Cluster 7, primarily composed of Lactocytes from dairy goats, exhibited elevated expression of LPL, a gene critical for cholesterol and lipid transport. Clusters 5 and 9 contained Lactocytes from all three species, with Cluster 5 enriched for genes involved in milk protein synthesis and Cluster 9 enriched for genes regulating milk fat production. Cluster 6 consisted of LHS cells from all species and stages, reflecting conserved hormonal response mechanisms in the mammary gland. Together, these results highlight that Lactocyte subtypes within Clusters 5 and 7 are evolutionarily conserved cell types in three species primarily responsible for milk protein and milk fat production across species (Figure 7E,F).

## 3 Discussion

The mammary gland is a highly dynamic organ composed of epithelial, stromal, and immune cells that together support milk production and tissue remodeling [80], undergoing cyclical remodeling across reproductive stages [44, 80]. Recent advances in single‐cell omics, particularly scRNA‐seq, have greatly improved our understanding of mammary gland cellular hierarchies and functional states across species [81, 82, 83]. While mouse models have been instrumental in uncovering mammary biology, accumulating evidence now highlights that ruminant mammary glands more closely resemble those of humans in both structure and function, establishing them as valuable models for studying epithelial differentiation and lactation [84, 85]. In this study, we employed snRNA‐seq and snATAC‐seq to profile the transcriptomic and chromatin accessibility landscapes of dairy goat mammary glands at the dry and lactation stages. Integrative analysis uncovered multiple epithelial subpopulations, including Lactocytes and LHS cells. Notably, Lactocytes were further subdivided into three distinct subtypes with different roles in milk component synthesis. We also identified a progenitor cell population termed LASP4 that was present at both two stages and that exhibited high plasticity and stem‐like characteristics. This observation is consistent with previous human studies suggesting that luminal epithelial progenitors can give rise to both Lactocytes and LHS cells [15]. Future studies are still needed to validate the differentiation capacity of LASP4 through in vitro functional experiments.

Spatial transcriptomic profiling of lactating mammary tissue confirmed the heterogeneity and organized structure, including lobular and alveolar structures, as well as the interstitium separating the lobules. We observed that LASP4 and Lactocytes2 tend to co‐enrich within the same lobular regions, whereas Lactocytes1 and Lactocytes3 are relatively concentrated in other regions. Notably, these lobules exhibit considerable variation in size. Previous studies have shown that mammary lobulo‐alveolar units during lactation display substantial structural and cellular heterogeneity. Lobule size can be influenced by the number of alveoli, the degree of alveolar expansion, and local milk accumulation, making variability in lobule size a common feature in histological sections [86]. Moreover, even during peak lactation, individual lobules may exist in different functional states, such as active secretion, cellular replenishment, or local remodeling, which can lead to differences in cellular composition [87]. In this context, LASP4 may represent a progenitor population contributing to the continuous replenishment of secretory epithelial cells, resulting in relative enrichment of progenitor‐like cells in some lobules, while others are dominated by fully differentiated secretory cells. In addition to epithelial lineages, immune cells represented a significant component, which supporting epithelial remodeling and alveolar development during lactation [88, 89, 90] and collaborating with epithelial cells to clear apoptotic cells during involution [88, 91, 92]. Plasma cells also contribute to lactation by secreting local IgA near alveolar structures, highlighting the importance of immune‐epithelial crosstalk in regulating mammary function [93].

To further link cellular regulatory landscapes with lactation traits, we integrated snATAC‐seq data with GWAS signals associated with milk production in dairy goats. This analysis revealed that lactation‐related SNPs were enriched in specific mammary cell types, suggesting cell‐type‐specific regulatory roles. For instance, SNPs within the DGAT1 locus were enriched in Lactocytes2, which are closely associated with milk fat synthesis, consistent with previous studies identifying DGAT1 polymorphisms as key determinants of milk fat percentage in goats [24]. We also discovered SNPs in the NPC1 locus enriched in LASP4 and all three Lactocyte subtypes. These findings also suggest that some genetic variants may influence milk yield indirectly by affecting the differentiation trajectory of progenitor populations such as LASP4. Meanwhile, we examined available eQTL data from goat mammary tissue and found that these candidate SNPs were not identified as significant eQTLs. The mechanisms by which genetic variants influence traits are diverse: In addition to affecting gene expression, SNPs may act by altering protein‐coding sequences, modulating splicing regulation, regulating non‐coding RNAs, or influencing chromatin accessibility. In this study, our snATAC‐seq data revealed that these SNPs are enriched in cell type‐specific chromatin‐accessible regions, suggesting that they may contribute to trait regulation by affecting chromatin states. However, these findings still require further functional validation. Due to the lack of a well‐established linkage disequilibrium (LD) reference panel in goats, regional LD among SNPs was not accounted for in this study, which may lead to an overestimation of enrichment signals in certain chromatin‐accessible regions. With the accumulation of goat population genomics data in the future, more accurate enrichment analyses could be performed using approaches such as LD score regression.

By integrating snATAC‐seq and GWAS of milk production traits, we further investigate the origin and differentiation trajectories of MECs. At the dry stage, the differentiation trajectory of LHS cells showed a marked expansion, which subsequently promoted the differentiation and maturation of Lactocytes. This process was accompanied by the restoration of alveolar lumen size, thereby initiating mammary gland renewal [94]. In contrast, at the lactation stage, one trajectory supported the maturation of LHS cells, which in turn drove the differentiation of LASP4 cells into functional Lactocytes [94]. Two additional trajectories supported milk secretion through Lactocytes differentiation. These findings are consistent with pseudotime analyses reported in humans, mice, and pigs. To identify key TFs driving lineage specification, we constructed GRN by integrating snATAC‐seq and snRNA‐seq data. Our analyses indicate that *EBF3* is a key TF regulating LHS cell differentiation at both the dry and lactation stages. At the dry stage, *EBF3* facilitates the differentiation of LASP4 and LASP2 into LHS cells, thereby reducing progenitor cell accumulation, initiating mammary gland renewal, and driving tissue remodeling. At the lactation stage, *EBF3* mainly supports LHS cell formation and the subsequent differentiation of LASP4 into functional Lactocytes to meet the demands of milk production. In comparison, *TFEB* shows a more stage‐specific role, predominantly acting to accelerates mammary gland renewal by promoting LHS cell differentiation at the dry stage. Transcriptomic analysis of GMECs following *EBF3* or *TFEB* knockdown further demonstrated that their target genes are enriched in pathways related to mammary gland development and cell differentiation, reinforcing their functional importance in LHS cell lineage specification. For Lactocytes differentiation, *THAP11* was identified as a central TF that may facilitate the transition from proliferative states (puberty and pregnancy) to secretory function by repressing C‐MYC [95] and regulating lactation‐relevant genes such as SLC52A2, which is involved in riboflavin transport and milk synthesis [96]. Notably, canonical pluripotency targets (e.g., OCT4, SOX2 and NANOG) were not enriched among *THAP11*‐regulated genes, suggesting that *THAP11* promotes secretory lineage specification via distinct mechanisms independent of classical stemness pathways [74]. Together, these findings identify *EBF3*, *TFEB*, and *THAP11* as candidate transcriptional regulators associated with LHS cell and Lactocyte differentiation, providing insights into the dynamic transcriptional programs underlying luminal epithelial lineage specification across different physiological stages.

In addition to TFs, stage‐specific cell‐cell communication also plays a critical role in regulating MEC differentiation. At the dry stage, AREG‐EGFR signaling within the EGF pathway is essential for ductal morphogenesis by promoting MEC proliferation [56]. At the dry stage, when the mammary gland undergoes structural regression, this signaling facilitates regeneration of the ductal network in preparation for the next lactation cycle. Additionally, by antagonizing epithelial apoptosis, it supports the remodeling of the ductal tree into a simplified structure [44, 97]. Consistent with previous studies, LHS cells may convert endocrine reproductive signals into paracrine or autocrine factors such as AREG to coordinate functional differentiation of mammary epithelial lineages [98]. At the lactation stage, IGF signaling emerged as a key regulator of mammary gland function [99]. Fibroblasts, *PDGFR*+ Fibroblasts and Lymphocytes secreted IGF, which acts on LHS cell via IGF1R to support milk production and lipid metabolism [100, 101]. Interestingly, IGF signaling was enriched in Lactocytes2 associated with milk fat production but was not detected in Lactocytes3, suggesting that precise regulation of IGF activity may be required for proper alveolar function. SPP1 was highly expressed at both the dry and lactation stages in mammary glands [90, 102]. During lactation, SPP1 secreted by LASP4 and Lactocytes3 may activate CD44 receptors and interact with IGF and EGF signaling to support milk secretion and ductal remodeling [103, 104], whereas at the dry stage, SPP1‐CD44 interactions predominantly contribute to ductal restructuring. The co‐expression patterns and predicted interaction strengths. These L‐R interactions were analyzed based on spatial transcriptomic data. However, the regulatory roles of these L‐R pairs remain require further experimental validation. We are currently optimizing a mammary organoid culture system, which will be used in future studies to functionally validate key candidate L‐R interactions. Overall, these findings highlight the complexity of stage‐specific signaling networks, in which LHS cells may serve as a critical hub integrating endocrine cues and local paracrine signals to coordinate lineage differentiation and mammary gland remodeling.

In summary, we generated an integrated single‐cell transcriptomic and epigenetic atlas of the goat mammary gland. By combining snRNA‐seq, snATAC‐seq and GWAS of lactation traits, we identified key epithelial subtypes, regulatory TFs and stage‐specific signaling pathways associated with mammary differentiation and remodeling. Notably, LHS cells and LASP4 play central roles in mammary renewal at the dry stage and milk synthesis during lactation, providing potential targets for improving milk production in ruminant livestock.

## 4 Conclusion

This study presents a comprehensive single‐cell transcriptomic and epigenetic atlas of the dairy goat mammary gland at the dry and lactation stages. By integrating GWAS and snATAC‐seq data, we linked non‐coding variants to specific cell types and milk production traits. Pseudotime analysis, GRN construction based on integrated snRNA‐seq and snATAC‐seq, and cell‐cell communication analysis revealed conserved and stage‐specific regulatory programs underlying epithelial differentiation between dry and lactation stages. Cross‐species comparisons further uncovered differential evolutionary rates of MECs, conserved milk‐producing subtypes, and lineage‐specific populations contribute to species‐specific milk composition. Together, these findings highlight the pivotal role of LHS cells in both mammary gland renewal during the dry stage and milk synthesis during lactation, providing a conceptual framework and potential targets for improving milk production in ruminant livestock.

## 5 Experimental Methods

### 5.1 Collection of Dairy Goat Mammary Tissues

All experimental procedures in this study were reviewed and approved by the Institutional Animal Care and Use Committee of Northwest A&F University. Mammary tissue samples were collected from three lactation and three dry‐stage Saanen dairy goats at a local slaughterhouse in Weinan, China. All goats had uniform body weight (approximately 35 ± 2 kg), and were fed ad libitum and singleton‐borne in a non‐pregnant state. The dry‐stage goats were about 21 months old (around 240 days postpartum) with 220 days of lactation, while the lactation‐stage goats were about 15 months old (around 90 days postpartum). Immediately after collection, mammary tissues were isolated and rinsed with pre‐chilled 1× PBS (Ambion, #AM9624), and placed on an ice‐cold plate for dissection. Each sample (excluding peripheral blood) was dissected into 5–10 pieces (50–200 mg each), rinsed thoroughly with 1× PBS, transferred to 1.5 mL cryotubes and embedded in optimum cutting temperature (OCT) compound (Sakura Finetek, #4583). Finally, samples were flash‐frozen in liquid nitrogen, stored for subsequent snRNA‐seq, snATAC‐seq and spatial transcriptomic analyses.

### 5.2 Single‐Nucleus Suspension Preparation

Single‐nucleus isolation was performed following previously established protocols [105, 106]. Briefly, frozen tissue samples were homogenized in 1 mL lysis buffer containing 250 mm sucrose (Ambion), 10 mg/mL bovine serum albumin (BSA, Ambion), 5 mm MgCl_2_ (Ambion), 0.12 U/µL RNasin Plus (Promega, #N2115) and 1 × Complete Protease Inhibitor Cocktail (Roche, #11697498001) using a homogenizer. To ensure uniformity, tissues underwent two additional rounds of homogenization. The homogenate was filtered through a 40 µm cell strainer, and nuclei were pelleted by centrifugation at 500 × g for 5 min at 4°C. The pellet was resuspended in 1 mL buffer containing 10 mg/mL BSA, 3 mm CaCl_2_, 10 mm Tris‐HCl, 320 mm sucrose, 0.1 mm EDTA, 2 mm magnesium acetate, 1 mm DTT, 1 × Complete Protease Inhibitor Cocktail and 0.12 U/µL RNasin. A final centrifugation at 500 × g for 5 min at 4°C was performed to pellet the nuclei. After obtaining purified nuclei, 4',6‐diamidino‐2‐phenylindole (DAPI) staining and microscopic examination were performed to assess nuclear integrity and purity, ensuring the absence of cytoplasmic residues or ruptured nuclei. If the homogenized samples contained abundant cellular debris or lipid droplets, additional purification was carried out using OptiPrep density gradient centrifugation.

Based on the initial cell count and the desired final concentration, nuclei were then resuspended in an appropriate volume of chilled Diluted Nuclei Buffer (10× Genomics, 2000153). The concentration of isolated nuclei was quantified using a Countess II FL Automated Cell Counter, and the nuclei were immediately processed for snRNA‐seq and snATAC‐seq library construction.

### 5.3 The Sample Preparation and Processing of snRNA‐seq Data

Raw sequencing reads generated by the Illumina platform in FASTQ format were pre‐processed using Trimmomatic software [107, 108]. The remaining high‐quality reads were retained as clean reads for subsequent analyses. Finally, FastQC [109] was used to assess the quality of the clean reads through basic quality control statistics.

Sample demultiplexing, barcode processing, and single‐cell 3′gene counting were performed using CellRanger (v7.1.0) [110]. snRNA‐seq reads were aligned to the ARS1 goat reference genome (GCA_001704415.1). Single‐cell data were processed using the R package “Seurat” (v.4.3.0) [111] from three input tables: genes, barcodes, and UMI counts. Initial data quality control was performed as follows: first, potential doublets were rigorously filtered out using the “DoubletFinder” function with an expected doublet rate of 0.3% (3 per 1000 cells) applied separately to the datasets from each stage. Following this, cells were retained for subsequent analysis only if they expressed between 200 and 7000 genes and had a mitochondrial transcript content below 5%. Data normalized and standardized were performed with the “NormalizeData”, “ScaleData” and “FindVariableFeatures” functions, identifying the top 2000 most variable genes. Dimensionality reduction and clustering were conducted using the Seurat workflow, including “RunPCA” (dims = 1:10), “RunUMAP” (dims = 1:10), “FindNeighbors” (dims = 1:10) and “FindClusters” (resolution = 0.3). Differential gene expression between clusters was performed using the “FindAllMarkers” and “FindMarkers” functions via the Wilcoxon rank‐sum test under parameters “min.pct = 0.25” and “logfc.threshold = 0.25”. *p*‐values were adjusted using the False Discovery Rate (FDR) method, with adjusted *p*‐values (*P*.adj<0.05) were considered statistically significant.

### 5.4 Iterative Clustering and Cell Type Annotation

Cell type annotation was performed through an iterative and hierarchical clustering strategy to systematically define both major lineages and fine‐grained subpopulations. The process was as follows: Broad‐Level Clustering (Level 0): We first performed an initial round of clustering on the integrated dataset at a moderate resolution to partition all cells into major, transcriptionally distinct classes. The identity of these broad classes was assigned based on the expression of canonical marker genes and their biological identities. Subclustering for Fine‐Grained Annotation (Level 1): To resolve cellular heterogeneity within each major lineage, we subset the data for each broad cell class and performed a second, independent round of analysis. For each subset, we re‐ran the entire dimensionality reduction and clustering pipeline, including “RunPCA”, “FindNeighbors”, “RunUMAP” and “FindClusters” functions, optimizing the clustering resolution for each subset to best capture distinct cell states. Differential expression analysis for these subclusters was performed using FindAllMarkers. Cell states within, for example, the epithelial compartment, such as Lactocytes1, Lactocytes2, Lactocytes3 and LHS cells, were confidently identified by cross‐referencing the top differentially expressed genes with established cell type‐specific signatures from the literature. Finally, cell types were annotated.

To further investigate the stage‐specific cellular composition, snRNA‐seq data from the dry and lactation stages were analyzed independently using the same pipeline, generating stage‐resolved single‐cell transcriptomic atlases. DEGs between two stages within the same cell type, as well as highly expressed genes in stage‐specific cell types, were further analyzed for functional enrichment using DAVID (https://davidbioinformatics.nih.gov/summary.jsp) [112] to perform KEGG pathway and GO enrichment analyses. The fgsea (v1.20.0) was used to perform GSEA analysis across different cell types.

### 5.5 Cell Cycle Regression Analysis

To evaluate and control for potential cell cycle effects in mammary cell types from dairy goats at the dry and lactation stages, we applied the standard cell cycle scoring and regression workflow implemented in the R package “Seurat”. Canonical S phase and G2/M phase marker gene sets were obtained from the Seurat *cc.genes* dataset. Each cell was then scored for S phase and G2/M phase activity using the “CellCycleScoring” function, which calculates S and G2/M scores and infers the most likely cell cycle phase.

To assess whether cell cycle effects contributed to transcriptomic variation, we performed principal component analysis (PCA) based on the expression of S phase and G2/M phase gene sets. Cells were clearly separated by cycle phase, indicating the presence of cycle‐related transcriptional variation. To minimize this confounding effect, S and G2/M scores were regressed out during the scaling step using the “ScaleData” function, ensuring that subsequent clustering and marker gene identification reflected true biological heterogeneity rather than cell cycle‐driven differences.

We further validated the correction by quantifying correlations between cell cycle phase assignments and clustering structure before and after regression, which showed a marked reduction in cycle‐associated bias. Finally, cells were visualized in two‐dimensional embeddings with color‐coded annotations according to their inferred cell cycle phases, providing an overview of cycling and non‐cycling cell distributions at both two stages. To avoid stage‐specific biases, the dry and lactation datasets were also analyzed independently using the same pipeline.

### 5.6 The Sample Preparation and Processing of snATAC‐seq Data

The nuclei suspension was loaded onto the Chromium Next GEM Chip H using 10 × Genomics reagents and barcoded with a 10 × Chromium Controller (10 × Genomics). DNA fragments from the barcoded nuclei were amplified, and sequencing libraries were constructed following the manufacturer′s instructions and using the Chromium NextGEM Single Cell ATAC Reagent Kits v1.1 (10 × Genomics). Library preparation was performed according to the Chromium NextGEM Single‐Cell ATAC Reagent Kits v1.1 User Guide RevC (10 × Genomics CG000209). The final libraries were pooled and sequenced on an Illumina NovaSeq PE50 platform using 2 × 50 paired‐end kits at Novogene.

The FASTQ raw sequencing data were converted into three matrix files: barcodes, matrix, and motif using cellranger‐atac mkfastq [113]. Reads were aligned to the ARS1 goat reference genome and quantified with cellranger‐atac count (10 × Genomics) using default parameters. The processed data were further analyzed with ArchR [114]. Arrow files were created using “createArrowFiles” function with the parameter “addGeneScoreMat = TRUE”, incorporate gene score matrix. Nuclei with transcription start site TSS enrichment scores≤4 or fragments≤1000 were filtered out. Potential doublets were predicted using “addDoubleScores” function and removed using the “filterDoublets” function. Next, dimensionality reduction was performed using the “addIterativeLSI” function, followed by clustering with Seurat's “FindClusters” function with parameters “resolution = 0.8”. UMAP visualization was fulfilled using the “addUMAP” function.

To integrate snATAC‐seq and snRNA‐seq data, the “addGeneIntegrationMatrix” function was applied, enabling transfer of cell type annotations and pseudo‐snRNA‐seq profiles to each snATAC‐seq cell. Peak calling for each cell type was performed using MACS2, and peak matrices were generated with “addPeakMatrix” function. During peak calling, the recommended parameters were used: –shift 100 –extsize 200 ‐q 0.05 to accommodate the sparsity of single‐cell ATAC data. Motif enrichment analysis was performed with “addMotifAnnotations” function with motifSet = “JASPAR2020”. Finally, MEC differentiation trajectories at both the dry and lactation stages were constructed using “addTrajectory” function, providing a foundation for subsequent GRN construction [115].

### 5.7 Spatial Transcriptomics Sequencing and Analysis

To ensure accurate morphological reference for spatial transcriptomic analysis, H&E staining was performed on frozen mammary tissue embedded in OCT compound. The procedure was as follows: fresh tissue blocks were embedded in OCT, rapidly frozen, and sectioned at a thickness of 10 µm using a cryostat. The sections were fixed with 4% paraformaldehyde, stained with hematoxylin, differentiation in acid alcohol, blued in ammonia water, and counterstained with eosin. Subsequently, sections were dehydrated through a graded ethanol series, cleared in xylene, and mounted with an aqueous mounting medium. All stained sections were examined under a microscope to confirm the integrity of tissue morphology before being used for spatial transcriptomic localization analysis.

Mammary gland tissues embedded in OCT compound were cryosectioned at a thickness of 10 µm. Preparation of the Stereo‐Seq chip and library were performed as described previously [116]. Tissue sections were adhered to a Stereo‐seq chip, fixed, and permeabilized. According to the standard Stereo‐seq protocol, reverse transcription and cDNA amplification were performed on the chip to construct spatially barcoded libraries. The resulting libraries were sequenced on a DNBSEQ‐T7 platform (MGI, China). Raw sequencing data were processed through the SAW pipeline to generate gene expression matrices, and spatial spots were aggregated into Bin 50 units for fundamental analysis. The resulting data in GEF format were used to confirm the presence and spatial localization of cell types previously identified by single‐cell transcriptomics. For downstream analysis, data were processed using Stereopy (v1.6.1) and converted into AnnData (h5ad) format for use with Scanpy (v1.10.3). Quality control included filtering out low‐quality spots and lowly expressed genes (minimum 200 genes per spot and minimum 500 counts per spot). The expression matrix was normalized and log‐transformed, followed by dimensionality reduction using PCA and construction of a neighborhood graph. UMAP was applied to visualize spatial patterns and identify distinct spatial clusters.

Spatial cell type deconvolution was performed using the cell2location (v0.1.3) [117]. A reference snRNA‐seq signature matrix was first constructed from the annotated goat mammary dataset comprising 14 cell types. The snRNA‐seq data were processed using Scanpy (v1.10.3) [118], and a regression model (cell2location.models.RegressionModel) was trained for 250 epochs on the reference data using cell type labels to infer cell type‐specific gene expression profiles. For spatial transcriptomic data, the raw counts and spatial coordinates were preprocessed and aligned with the same gene annotation. The trained reference model was then used to deconvolve the spatial transcriptomic spots using the Cell2location model, which was trained for 30,000 epochs to estimate the posterior distributions of cell type abundances at each location. Low‐confidence predictions were filtered out by retaining only cell types whose fifth percentile of the posterior distribution (Q05) exceeded 0 cells per spot and whose maximum Q05 across all spots was ≥ 2 cells, ensuring robust spatial mapping. The resulting cell type abundance estimates (means and 95% credible intervals) were visualized in spatial context using the cell2location plotting utilities, with spot size and transparency adjusted for clarity. All analyses were conducted in Python (v3.10.12). Spatial transcriptomics data were analyzed using SpaGene (v0.1.0) to investigate L‐R pairs. A spatial neighborhood network was constructed based on gene expression and spatial coordinates, and spatial correlations between genes were calculated to assess LR co‐expression. The results were visualized using the plotLR function.

### 5.8 Immunofluorescence Staining

Paraffin‐embedded mammary gland tissue sections were dewaxed, rehydrated, and subjected to heat‐induced antigen retrieval. Briefly, sections were sequentially treated with xylene I and xylene II (15 min each), followed by a graded ethanol series (100%, 95%, 85% and 75%, 5 min each) and finally rinsed in deionized water. The slides were then immersed in citrate buffer (pH = 6.0) for antigen retrieval and heated in a pressure cooker for 2 min after steaming, followed by natural cooling to room temperature. To quench endogenous peroxidase activity, the sections were incubated with 3% H_2_O_2_ at room temperature for 20 min. Afterward, the sections were blocked with 10% normal goat serum at 37°C for 30 min to prevent nonspecific binding. Following blocking, the slides were incubated overnight at 4°C with appropriately diluted primary antibodies (Table S14). The next day, the sections were thoroughly washed with PBS (pH = 7.4) and then incubated with species‐matched fluorescent secondary antibodies for 1 h at room temperature in the dark. After washing, nuclei were counterstained with DAPI for 5 min. Finally, all sections were rinsed with PBS, mounted with an anti‐fluorescence quenching mounting medium, and imaged using a fluorescence microscope.

### 5.9 Enrichment Analysis of Lactation‐Associated SNPs in Cell Type‐Specific Chromatin Accessibility Regions

To investigate the enrichment of lactation‐associated SNPs within cell type‐specific chromatin accessibility regions of the mammary gland, we first identified cell type‐specific accessible chromatin regions based on snATAC‐seq data. A total of 245 SNPs significantly associated with six milk production traits, including MY, FP, PP, FY, PY and SCS (*p* <5 × 10^−^^8^) were collected from published studies, and their genomic coordinates and associated traits were recorded. These SNPs were then mapped to the chromatin accessibility regions of each cell type to construct a cell type‐trait SNP count matrix. Enrichment of trait‐associated SNPs within the accessible regions of each cell type was assessed using Fisher's exact test, and odds ratios and corresponding *P*‐values were calculated. To control for false positives arising from multiple testing, *P*‐values were adjusted using the Benjamini‐Hochberg method, with a significance threshold set at FDR < 0.05. All statistical analyses were performed in R.

### 5.10 CytoTRACE Stemness Analysis and Slingshot Pseudotime Analysis of MECs

CytoTRACE (v0.3.3) [119] provides a powerful framework for assessing cellular differentiation potential based on gene counts, significantly improving the evaluation of cell differentiation at the single‐cell level. Unlike conventional lineage trajectory inference methods, CytoTRACE predicts the relative differentiation state and direction of individual cells without relying on a predefined developmental time scale or continuous trajectories. In this study, CytoTRACE was utilized to calculate stemness scores of MECs at both the dry and lactation stages, providing insights into the differentiation potential and hierarchical organization of epithelial cells at different stages. The analysis first input the Seurat‐normalized expression matrix and ran the CytoTRACE algorithm with default parameters. The calculated stemness scores were then mapped to UMAP maps to visually demonstrate the differential distribution of differentiation states across different cell subpopulations. In addition, to more intuitively illustrate the relationship between cellular differentiation potential and subpopulation identity, we integrated the cell type annotation results to compare the distribution of CytoTRACE scores across different subpopulations and plotted the trend of differentiation potential along the pseudotime trajectory.

Furthermore, Slingshot [120] was utilized to analyze the differentiation trajectories of MEC at both stages based on the results of CytoTRACE differentiation potential analysis. Slingshot is a computational tool that integrates low‐dimensional spatial embeddings to infer potential lineage structures based on intercellular spatial relationships and to perform pseudotime ordering and dynamic estimation of gene expression within different lineages. In this study, we used the Seurat clustering results as the input for Slingshot and selected the cell population with the highest LASP4 expression, according to the CytoTRACE analysis results, as the starting point for pseudotime analysis to automatically fit potential differentiation trajectories. Through Slingshot analysis, we systematically characterized the temporal dynamics and lineage relationships of MECs at the dry and lactation stages, annotating key bifurcation points and potential intermediate cells between different lineages. Finally, we integrated differential gene expression and functional enrichment analyses to comprehensively investigate the key gene expression changes and corresponding biological pathways along different differentiation trajectories, thereby revealing the regulatory patterns underlying the shifts in differentiation direction and functional states of MECs between the dry and lactation stages.

### 5.11 Construction of the GRN for the Trajectories of Epithelial Cells

To construct a GRN, it is essential to map cells across different modalities (RNA and ATAC), enabling the association of TF/gene expression with gene/TF activity and chromatin accessibility at the single‐cell level. Therefore, scMEGA (v0.2.0) was used with default parameters to construct the GRN [121]. First, cells were projected into the shared co‐embedded space using canonical correlation analysis (CCA) implemented by Seurat, where features include gene expression from snRNA‐seq and gene activity scores from snATAC‐seq. Subsequently, “scMEGA” utilizes the OptMatch pairing method to perform one‐to‐one cell matching between snRNA‐seq and snATAC‐seq data, ensuring precise correspondence between different modalities and generating a cross‐modal or pseudo‐multimodal dataset that serves as the foundation for subsequent GRN inference.

Based on the resulting multimodal or pseudo‐multimodal data, scMEGA identifies candidate TFs and target genes. For MECs at the dry and lactation stages, pseudotime trajectories characterizing potential dynamic processes are inferred using a supervised method implemented in ArchR. scMEGA then estimates TF binding activity in each cell by integrating chromatin accessibility profiles. To identify active TFs, scMEGA calculates both TF binding activity and TF expression levels, and selects TFs that are active in both as candidate regulatory factors. To quantify the regulatory importance of TFs, we constructed a directed network based on the regulatory relationships inferred by scMEGA and calculated the PageRank score (global influence) and betweenness centrality (network bridging role) for each TF. After Z‐score normalization of both metrics, a composite importance score was calculated as the Euclidean distance between each TF and the minimum value point in the normalized two‐dimensional space. Subsequently, scMEGA computes gene expression dynamics along the pseudotime trajectory and selects the top 10 most variable genes as trajectory‐related target. Finally, scMEGA uses ArchR functions to associate the selected genes with peaks based on the correlation between gene expression and peak accessibility at the single‐cell level.

To construct the GRN, scMEGA quantifies the correlation between TF binding activity and the expression of candidate target genes. It further refines regulatory links by extracting enhancer‐to‐gene associations from the predicted peak‐to‐gene relationships and incorporating TF binding site predictions from chromVAR. A gene is considered a target of a TF only if it is associated with at least one enhancer and the TF binds to that enhancer, yielding an enhancer‐based GRN. Finally, scMEGA integrates the quantitative GRN and enhancer‐based GRN, retaining only high‐confidence TF‐gene regulatory interactions supported by both networks. These interactions are weighted by their correlation strength, and the final directed, weighted GRN is visualized using the R package “igraph” [122], thereby revealing the network reconfiguration and key regulatory factor dynamics underlying the transcriptional regulation of MECs at the dry and lactation stages.

### 5.12 Preparation and Culture of GMECs

The GMECs were isolated from healthy dairy goats following previously described procedures [123]. Mammary tissue samples were collected and repeatedly rinsed with D‐Hank's solution containing 3% penicillin‐streptomycin (Pen‐Strep, Solarbio, Beijing, China) to remove residual blood and debris. The parenchymal tissue was minced into approximately 1 mm^3^ in size and placed in dishes pretreated with fetal bovine serum (FBS, Gemini, USA). Cells were cultured in DMEM/F12 medium (Hyclone, Shanghai, China) supplemented with 5% FBS, 1% Pen‐Strep, 1% insulin (Solarbio, Beijing, China), 2% hydrocortisone (Sigma, Shanghai, China), and 0.1% epidermal growth factor (EGF, Invitrogen, Carlsbad, CA, USA). When cell confluence reached 80%–90%, the cells were digested with 0.25% Trypsin‐EDTA (0.25% trypsin and 0.02% EDTA, Solarbio, Beijing, China) at 37°C for 5 min. The cell suspension was then collected and centrifuged at 1000 × g for 4 min. The obtained cells were resuspended in growth medium and maintained at 37°C in a humidified incubator with 5% CO_2_. The culture medium was refreshed every 24 h.

### 5.13 Transfection of GMECs With siRNA and Detection of Target Gene Expression

Gene silencing was performed using siRNAs targeting *EBF3* and *TFEB* (siRNA‐*EBF3* and siRNA‐*TFEB*), with the specific sequences listed in Table S15. When GMECs reached approximately 40%–60% confluence, cells were transfected with 50 nm siRNA‐NC, siRNA‐*EBF3* or siRNA‐*TFEB* using Lipofectamine 2000 according to the manufacturer′s instructions. Transfected cells were harvested after 24 h for RT‐qPCR and RNA‐seq analyses, and after 48 h for western blotting analysis.

Total RNA was extracted using Trizol reagent (Takara, Dalian, China) following the manufacturer's protocol. RT‐qPCR was performed using the TB Green Kit (Takara, Dalian, China), with glyceraldehyde‐3‐phosphate dehydrogenase (*GAPDH*) serving as the internal control. Relative gene expression levels were calculated using the 2^−ΔΔCq^ method. The primer sequences used for RT‐qPCR are provided in Table S16. For protein analysis, cells were lysed using protein lysis buffer, and total protein was extracted for western blotting. GAPDH was used as the internal loading control, and protein abundance was analyzed using Image J software. Detailed information on the antibodies used for western blotting is provided in Table S14.

### 5.14 Statistical Analysis

All data were generated using GMECs of three goats to do three biological replicates. The significance analysis performed via unpaired *t*‐test between two group comparisons (NC vs. siRNA‐*EBF3* and NC vs. siRNA‐*TFEB*). For all analyses, *p* <0.05 was considered statistically significant. The data are presented as the means ± SEM, and GraphPad Prism 6.0 software were used to analyze difference.

### 5.15 Collection of GMECs Treated With siRNA and RNA‐seq Analysis

Total RNA was extracted from siRNA‐treated cells using Trizol reagent following the manufacturer's instructions. RNA integrity was then assessed using an Agilent 2100 Bioanalyzer (Agilent Technologies).

Total RNA quality was first assessed, and poly(A)+ mRNA was enriched using Oligo(dT) magnetic beads. The purified mRNA was fragmented using Fragmentation Buffer, followed by first‐ and second‐strand cDNA synthesis. The resulting double‐stranded cDNA was purified, end‐repaired, A‐tailed, and ligated to sequencing adapters. cDNA fragments of approximately 350 bp were selected and amplified by PCR to generate the cDNA library. Finally, the double‐stranded cDNA was denatured to single‐stranded DNA, and libraries were formed using circularization primers. The insert size of the library was detected by Qubit3.0 fluorometer and an Agilent 2100 bioanalyzer. High‐throughput sequencing was performed on the DNBSEQ‐T7 platform. The obtained raw reads were cleaned to remove adapters and low‐quality reads using fastp (v0.19.7) [124]. Clean reads were aligned to the goat reference genome (ARS1) using HISAT2 (v2.2.1) [125]. Gene‐level read counts were quantified with the featureCounts function in the Subread package (v2.0.8).

The DEGs were analyzed using the R package “DEGSeq” (v1.34.1) based on read counts. Genes with an absolute |Log_2_FoldChange| ≥ 1.5 and an adjusted *p* <0.05 were considered significantly differentially expressed. GO and KEGG pathway enrichment analyses of upregulated and downregulated DEGs were subsequently performed using the DAVID database to explore their potential biological functions.

### 5.16 Cell–Cell Communication Analysis

Cell–cell communication networks were inferred using the CellChat R package (v1.6.1) based on ligand‐receptor interactions [126]. For dry and lactation stages, the normalized gene expression matrices and cell‐type annotations derived from the snRNA‐seq data were used as input. The CellChatDB database was applied as the reference for known ligand‐receptor pairs. Data preprocessing included identification of overexpressed ligands and receptors, computation of communication probabilities, and filtering of significant signaling interactions (*p*<0.05). Communication networks were visualized using hierarchical and circular plots to depict both global and pathway‐specific interactions. Comparative analyses between stages were performed using the “compareInteractions” and “netVisual_diffInteraction” functions to identify stage‐specific signaling pathways and dominant sender‐receiver relationships. The inferred networks were further summarized at the signaling pathway level to highlight key regulators and intercellular signaling shifts associated with lactation.

### 5.17 Cross‐Species Comparisons of Single‐Cell Transcriptomic Data

First, scRNA‐seq data from the mammary glands of human [127], mice [128], pigs [14], cows [129], and cynomolgus monkeys [130] were collected and processed using the same Seurat parameters. To facilitate cross‐species integration, 1:1 orthologous gene were identified using eggNOG [131]. Subsequently, Harmony was applied to integrate the multi‐species datasets and generate combined cell clusters. The average gene expression within each cluster was calculated. DEGs across clusters and species‐specific genes within each cluster were identified. To further characterize evolutionary patterns, scCoGAPS [122] was used to decompose gene expression profiles into distinct gene expression modules for each species. The projectR tool was then employed to map other species into these modules, identifying convergent and divergent gene expression patterns across species. Shared epithelial cell types across different species were annotated, and MetaNeighbor analysis was performed to find homologous cell types that exhibit functional innovations in different species, thereby constructing a mammary tissue cell type correspondence network.

Next, the evolutionary rate analysis of mammary tissue cell types was performed. The snRNA‐seq data from each species was re‐annotated and classified into major cell classes. For each species, cells from corresponding major classes were subsampled at different proportions, and a pseudo‐bulk transcriptomic strategy was applied to calculate the correlations among these cells. This process was repeated 100 times to minimize batch effects. The resulting correlation matrices were processed to compare the evolutionary trajectories of each cell type to species divergence time. Finally, a collection of genes related to milk composition were compiled, and the expression levels of these genes across multiple species were examined to assess the potential of each species for producing high‐quality milk.

## Author Contributions

**Jun Liu** and **Yu Wang** conceived and designed the project. **Xiaoru Yan**, **Xiaoyu Mi**, **Guanghui Tan**, **Xinmei Li**, and **Zhenliang Zhu** performed single‐cell transcriptomic and chromatin accessibility analyses. **Zhenyu Wei**, **Huimei Fan**, **Yamei Wu**, **Tao Shi** helped with bioinformatic analyses. Xiaoru Yan, Xiaoyu Mi, Guanghui Tan, and Xinmei Li provided critical intellectual input and data interpretation. **Lingzhao Fang** and **Yuanpeng Gao** provided useful feedback and discussions. Jun Liu provided funding, discussion, and supervision. Xiaoru Yan, Yu Wang and Jun Liu prepared the manuscript with input from all authors.

## Funding

This work was supported by the Major Agricultural Biological Breeding Project (Grant No. 2022ZD04014) and the National Key Research and Development Programme of China (Grant No. 2021YFF1001000).

## Conflicts of Interest

The authors declare no conflicts of interest.

## Supporting information




**Supporting File 1**: advs76753‐sup‐0001‐FigureS1‐S8.docx.

**Supporting File 2**: advs76753‐sup‐0002‐TableS1‐S16.xlsx.

## Data Availability

FASTQ files and processed data are available from the corresponding author upon request.
