# Supplementary material for: Integrated Single‐Nucleus Multi‐Omics Atlases Reveal Lineage Plasticity and Regulatory Networks of Luminal Epithelial Cells During Mammary Gland Lactation and Involution
Source: Adv Sci (Weinh). 2026 Jul 27;13(58):e76753. doi: 10.1002/advs.76753 (PMC13403729; doi:10.1002/advs.76753)
Supplement: Supplementary file 1 [file ADVS-13-e76753-s002.docx]

**Figure S1. Major mammary epithelial, stromal and immune cell types identified by snRNA-seq.** **(A, E)** UMAPs of snRNA-seq, mammary cells colored by cell class at the dry (A) and lactation (E) stage, respectively. Different colors represent different cell types. **(B, F)** Feature plot of cell class-specific markers for each cell class as identified by multi-pairwise differential gene expression analysis of snRNA-seq data at the dry (B) and lactation (F) stage, respectively. **(C, G)** Heatmap displaying the top5 specifically expressed genes in each cell type uniquely present in mammary gland at the dry (C) and lactation (G) stage, respectively. **(D, H)** Spearman′s correlation analysis of gene expression profiles among the cell types at the dry (D) and lactation (H) stage, respectively. Vas & lymph, Vascular and lymphatic endothelial cells. **(I)** Cell proliferation regression analysis of annotated cell types in mammary gland tissues at the dry and lactation stages. Left: dry stage; Right: lactation stage. **(J)** UMAP plots showing the distribution of mammary gland cells from each stage. Different colors represent different stage.

**Figure S2. Comparative transcriptomic and functional analysis of mammary gland cell populations between the dry and lactation stages. (A)** The left figure represents the differential gene expression analysis of gene expression profile in the same cell types between the dry and lactation stage, with the plotted points indicating genes upregulated in the lactation stage relative to the dry stage. The right figure represents the GO enrichment of upregulated DEGs，with the displayed genes corresponding to those enriched in different GO terms. Different colors represent different cell types. **(B)** Differential expression analysis of stromal and immune cells in the mammary gland between the dry and lactation stages. Genes shown at the top are significantly upregulated DEGs, while genes shown at the bottom are significantly downregulated DEGs. **(C)** GO enrichment analysis of DEGs upregulated in stromal cells during lactation compared with the dry stage. **(D)** GO enrichment analysis of DEGs upregulated in immune cells during lactation compared with the dry stage. **(E)** GO enrichment analysis of specifically expressed genes in cell types uniquely present in mammary glands of the lactation stages. Vas endo cells, Vascular endothelial cells; Lym endo cells, lymphatic endothelial cells; *PDGFRA*+ fibro, *PDGFRA*+ fibroblast; DEG, Differential gene expression. **(F)** GSEA enrichment analysis of Lactocytes1 and Lactocytes2. C2 and C5 represent the curated gene sets from the C2 and C5 collections in the msigdbr database used for GSEA analysis. NES denotes the normalized enrichment score (Normalized Enrichment Score), where a larger absolute value of NES indicates a higher degree of enrichment.

**Figure S3. The spatial transcriptomic analysis and immunofluorescence (IF) staining of different epithelial subpopulations in goat mammary gland tissue. (A)** Unsupervised clustering of spatial transcriptomic data from the lactating mammary gland tissue, with different colors representing different clusters (n = 10). Each irregular shape represents a spot after cell segmentation. **(B)** Cell2location mapping of mammary gland tissue sections reveals the spatial matching of annotated cell types to their expected anatomical structures. **(C)** IF staining of different epithelial subpopulations in goat mammary gland tissue. Shown are IF results for LASP4, LHS cells, Lactocytes1, Lactocytes2 and Lactocytes3 (scale bar = 50 μm). Blue indicates DAPI staining, while red and green correspond to the specific marker genes for each respective cell type.

**Figure S4. Genes dynamically regulated along pseudotime.** **(A-B)** Heatmap of genes exhibiting pseudotime-dependent changes along the differentiation trajectory of Dry-Lineage1 (progresses from LASP4 to LASP2 and then to LHS cell) and Lactation-Lineage1 (progresses from LASP4 to Lactocytes2 and then to LHS cell). **(C)** Highlighting hormone receptor genes exhibiting pseudotime-dependent changes along the differentiation trajectory of dry and lactation stage. **(D)** Highlighting lactation-related genes exhibiting pseudotime-dependent changes along the differentiation trajectory of dry and lactation stage, respectively. **(E)** Highlighting other four genes exhibiting pseudotime-dependent changes along the differentiation trajectory of dry and lactation stage, respectively.

**Figure S5. Endogenous Biological Function Validation of *EBF3* and *TFEB*. (A)** Immunofluorescence co-localization of the core transcription factors (TFs) *EBF3* and *TFEB* with marker proteins of distinct cell types along the Dry- and Lactation-Lineage1 differentiation trajectories. Immunofluorescence staining revealed that, along the Dry-Lineage1 trajectory, EBF3 and TFEB were co-localized with marker proteins of LASP2 (*KRT8* and *ALDH1A3*) and LASP4 (*KRT8* and *KIT*), respectively. Along the Lactation-Lineage1 trajectory, EBF3 and TFEB showed co-localization with marker genes of LASP4 (*KRT8* and *KIT*) and Lactocytes2 (*KRT8* and *PLIN2*), respectively. **(B)** The original images of Western blot. The units are KDa. siRNA-NC, negative control; siRNA-*EBF3*, *EBF3* knockdown group; siRNA-*TFEB*, *TFEB* knockdown group.

**Figure S6. Cell-cell communication across all annotated cell types. (A, H)** Cell types exhibiting the highest probabilities as senders or receivers of distinct signaling pathways at the dry (A) and lactation stages (H). **(B-F)** Hierarchical plot shows the inferred intercellular communication network for BMP, FGF, EGF, NRG and SPP1 signaling at the dry stage. This plot consists of two parts: Left and right portions highlight the autocrine and paracrine signaling to epithelial states and to other non- epithelial cell states, respectively. Solid and open circles represent source and target, respectively. Circle sizes are proportional to the number of cells in each cell group and edge width represents the communication probability. Edge colors are consistent with the signaling source. **(G)** The signaling pathways of dry and lactation stage were ranked based on their pairwise Euclidean distance in the shared two-dimensional manifold, respectively. Larger distance implies larger difference. **(I-L)** Hierarchical plot shows the inferred intercellular communication network for BMP, IGF, TGFβ and SPP1 signaling at the lactation stage. This plot consists of two parts: Left and right portions highlight the autocrine and paracrine signaling to epithelial states and to other non- epithelial cell states, respectively. Solid and open circles represent source and target, respectively. Circle sizes are proportional to the number of cells in each cell group and edge width represents the communication probability. Edge colors are consistent with the signaling source. Vas endo cells, Vascular endothelial cells; Lym endo cells, lymphatic endothelial cells; *PDGFRA*^+^ fibro, *PDGFRA*^+^ fibroblast; *TOP2A*^+^ macro, *TOP2A*^+^ macrophage.

**Figure S7. Spatial distribution of L-R pairs interactions in the dairy goat mammary gland.** **(A-D)** Visualization of IGF1-IGF1R (A), TGFB2-TGFBR1 (B), SPP1-CD44 (C), and SPP1-ITGB6 (D) interactions. The left panels show the expression levels of ligands and receptors, and the right panels indicate interaction strength. **(E-F)** Visualization of BMP6-ACVR2A/BMPR2 interactions. Different colors represent the expression levels of ligands and receptors.

**Figure S8. Cell-cell communication between other cell types and epithelial cells at different stages. (A)** Comparison of the significant ligand-receptor pairs between dry and lactation stage, which contribute to the signaling from stromal and immune cell to mammary epithelial cells (MECs) and stromal cells. **(B)** Comparison of the significant ligand-receptor pairs between dry and lactation stage, which contribute to the signaling from stromal and immune cell to immune cells. Dot color reflects communication probabilities and dot size represents computed *P*-value. Empty space means the communication probability is zero. *P*-values are computed from one-sided permutation test. Vas endothelial cells, Vascular endothelial cells; Lym endothelial cells, lymphatic endothelial cells.
